# Supplementary material for: Dietary restriction and the transcription factor clock delay eye aging to extend lifespan in Drosophila Melanogaster
Source: Nat Commun. 2022 Jun 7;13:3156. doi: 10.1038/s41467-022-30975-4 (PMC9174495; doi:10.1038/s41467-022-30975-4)
Supplement: Supplementary file 1 — Supplementary Information [file 41467_2022_30975_MOESM1_ESM.pdf]

Supplementary Discussion 1.

DR amplifies and modifies circadian transcriptional output

DR and DR-memetics have long been known to improve circadian behavioral rhythms in old age[1]. Over the past decade, improvements in molecular genetic techniques and next-generation sequencing have allowed investigators to examine how nutrient composition and time of feeding influence circadian transcriptional rhythms. Reports in mammals have demonstrated that calorie restriction, a reduction in total calorie intake without malnutrition, enhances the number and amplitude of rhythmic transcripts[2]. Inversely, high-nutrient diets, such as high-fat/western diets suppress circadian transcriptional rhythms[3]. The near doubling in the number of circadian transcripts we quantified on DR vs AL in *Drosophila* is consistent with observations in mammals. Additionally, transcripts oscillating on DR displayed an increased circadian amplitude. DR-mediated increases in the number of circadian transcripts, and their amplitude, is likely due to enhanced transcriptional output by CLK/CYC. Recent reports in both mice and flies have demonstrated that nutrient-sensing mechanisms (i.e., AMPK/TOR and Sirtuin signaling) signal directly to the core-clock transcription factors to activate transcription[4-6]. For instance, the *Drosophila* AMPK, which is activated in response to cellular energy depletion (e.g., elevated AMP concentrations), directly phosphorylates CLK, enhancing its circadian transcriptional output[4]. Because we extracted mRNA from populations of flies, the transcript expression values we report here are influenced by both individual and population-wide transcript expression levels. Therefore, the DR-mediated improvements to the circadian transcriptome we have observed may also reflect greater synchronicity between individual flies.

Interestingly, we also observed relatively low overlap between the transcripts that oscillate on AL compared to DR. We found that DR-oscillating genes are enriched for

processes related to homeostatic function, while circadian processes on AL-oscillating genes are enriched for processes related to damage-response pathways. These findings are similar to those reported in mouse liver tissue, where there was relatively small overlap in circadian transcripts comparing the transcriptomes of mice reared on standard chow versus those on calorie restriction[2]. A combination of aging and damage response signals (e.g., reactive oxygen species) also influence which transcripts cycle in the fly[7]. This phenomenon, termed "circadian reprogramming," is also observed in response to nutrient cues, where differing nutrient signals direct which specific transcripts are transcriptionally targeted downstream of the molecular clock. The similarities between the diet-dependent changes we report here and those previously reported in mice on calorie restriction indicates that the molecular clock's response to nutrient restriction is evolutionarily conserved.

Given DR's ability to robustly extend lifespan while amplifying circadian transcriptional output, we postulated that DR-sensitive circadian processes play an important role in slowing aging and improving survival. Although highly informative, to date, the diet-dependent circadian transcriptome studies have analyzed only a small number of mammalian tissues and thus have provided only a limited description of how diet influences circadian transcriptional output at the whole-organism level. Our ability to analyze the AL/DR circadian transcriptomes in the whole fly allowed for an unbiased approach for identifying the most DR-sensitive, cyclic processes throughout the body. This approach led to the observation that phototransduction was among the top circadian processes amplified by DR. The phototransduction genes we identified were also cyclic in flies reared on AL, albeit at a lower expression and circadian amplitude, indicating that their transcriptional regulation is likely not a result of circadian reprogramming. This, however, highlights the biological importance of their circadian regulation. A limitation

of our AL/DR circadian transcriptome analyses is that they are likely under-powered to identify the full spectrum of eye-specific circadian transcripts, because our mRNA samples were pooled from whole-body lysates and were collected for only one circadian cycle (24hr). Analyses of a more robust circadian transcriptome, performed from mRNA collected from heads, over 2 circadian cycles (48hr), indicated that phototransduction components were among the most rhythmic circadian processes, thus underscoring the importance of circadian regulation within the eye[7].

DR delays visual senescence by amplifying circadian rhythms in the eye

Metabolic dysfunction is strongly correlated with accelerated aging and eye-disease in mammals (e.g. diabetic retinopathy)[8,9]. Declines in the circadian amplitude of clocks within the eye have been reported in wild-type mice with age and in models of diabetic retinopathy, which may further exacerbate disease pathology[10,11]. Calorie restriction protects against several age-related eye diseases—dry-eye disease, cataracts, and age-related macular degeneration[12]. Calorie restriction also has a neuroprotective effect in photoreceptors and retinal-ganglion cells with age[12]. To date, no studies have investigated whether calorie restriction enhances circadian amplitude within the eye or whether its benefits within the eye are dependent on the molecular clock (in flies or mammalian models). Our results in flies demonstrate that DR amplifies circadian rhythms within the eye and delays visual senescence in a CLK-dependent manner. Additionally, we identified the DR-sensitive CLK-output genes *Gβ76c*, *retinin*, and *sun* and demonstrated that their knockdown in the eye accelerated visual declines, thus indicating that DR's neuroprotective role in the eye functions mechanistically through the molecular clock.

Several age-associated morphological and physiological declines have been reported in circadian mutant mouse models[13]. The positive-limb of the core molecular clock in mice is comprised of the basic-helix-loop-helix transcription factors BMAL1 and CLOCK[14]. Mice harboring whole-body genetic knockouts of either BMAL1 or CLOCK develop cataracts and corneal inflammation with age[13]. Additionally, photoreceptor-specific (cone-cell, HRGP-Cre x *Bmal1* fl/fl) BMAL1 knockout mice display a significantly altered circadian transcriptome, a shift in the distribution of short vs medium wavelength opsins, and a reduction in photoreceptor cell viability with age[15,16]. Consistently, our data demonstrates that diminishing CLK function in adult animals (post-development) is sufficient to drive eye aging in flies. However, there are important distinctions between the mechanism of phototransduction used by mammalian rod and cone photoreceptors, and what exists in the fly.

In mammals, light-activated rhodopsin in rod and cone photoreceptor neurons couples to, and inactivates, cyclic nucleotide gated channels, hyperpolarizing the cell[17]. This is distinctly different from what occurs in the fly, where light-activated rhodopsin couples to a TRP channel, which when activated depolarizes the cell[18]. However, in a third class of mammalian photoreceptors, the intrinsically-photosensitive retinal ganglion cells (ipRGCs), there is a nearly identical mechanism of phototransduction to *Drosophila*[19]. The ipRGCs play a role in non-image forming light sensation, effecting pupillary constriction and the entrainment of the central circadian clock to light. There is some evidence that eliminating *Bmal1* in mice (either specifically in their ipRGCs or throughout their entire body) impairs the functionality of the ipRGCs[20]. This is consistent with what we observed when we disrupted *clk* in the *Drosophila* photoreceptors. Together, this suggests that there may be a conserved mechanism through which circadian clocks mediate the health of photoreceptor cells.

An inability to adequately respond to light stress may underly the accelerated photoreceptor aging we observe when CLK function is diminished in the eye of adult flies. Chronic exposure to phototoxic wavelengths or strong ambient light intensities, as well as mutations in light adaptation proteins, elevates intracellular calcium ion concentrations that result in rapid photoreceptor degeneration[21,22]. Pittendrigh's "escape the light" hypothesis posits that circadian rhythms evolved as a means for cells/organisms to anticipate and manage the deleterious effects of daily light exposure[23,24]. One of the key neuroprotective functions of intrinsic clocks within photoreceptors is their ability to modulate time-of-day sensitivities to light. Electroretinogram (ERG) recordings in both flies and mammals have revealed a circadian response pattern that peaks at night when luminescence is approximately one-billion-fold less than during the day, and this pattern in light sensitivity is abolished in in circadian mutants[25,26]. Interestingly, exposing rats to a bout of intense light at night results in significantly greater photoreceptor damage and degeneration than when the same treatment is performed during the day, thus highlighting the physiologic importance of the clocks in suppressing light sensitivity during the day[27]. Our acrophase analyses revealed that circadian transcripts that promote photoreceptor activation (Ca^{2+} influx) reach peak expression during the dark-phase, while genes that terminate the phototransduction response (i.e., reducing rhodopsin mediated signaling) peak in anticipation of the light-phase (Fig. 1f and Supplementary Fig. 1j).

The eye regulates longevity in Drosophila

With age, declines in tissue homeostasis and chronic activation of the immune system increases local and systemic inflammation, termed "inflammaging." The deleterious effects from inflammaging exacerbate pre-existing aging phenotypes and reduce survival[28,29]. Interestingly, partial inhibition of the primary immune response regulator,

NFkappaB, extends lifespan in *Drosophila* [30]. Photoreceptor degeneration is a main source of inflammation within the mouse retina[31]. Here, our results demonstrate that diminishing neuronal CLK function and forcing photoreceptor degeneration significantly elevates systemic immune responses. Furthermore, we report dampened AMP expression in the bodies of Rh null lines, indicating that reductions in phototransduction coincide with reduced systemic inflammation in the fly. We have also found that flies reared on DR, which improves photoreceptor viability, displayed dampened immune responses in comparison to flies reared on AL. Interestingly, photoreceptor degeneration caused by light- or calcium-mediated excitotoxicity is primarily the result of necrotic cell death[21]. Forced photoreceptor necrosis also results in necrotic cell death of surrounding cells[32]. Given that cytosolic f-actin can drive the sterile immune response in the fly, it is possible that the increased systemic inflammation we report is due in part to elevations in necrosis[33]. However, future studies will be needed to elucidate how diet and circadian rhythms influence necrotic cell death in photoceptors, and the effect this has on the local niche.

Circadian disruption, achieved either genetically or via chronic circadian misalignment with the environment, is associated with reduced longevity[34,35]. Long-lived humans (i.e., centenarians) display significantly improved behavioral rhythms compared to “normal” aging groups[36]. Inversely, studies in mice and flies have demonstrated that organisms that display arrhythmic, or non-24h rhythms, are significantly shorter-lived than those who display near 24h (wild-type) circadian rhythms[37,38]. Furthermore, chronic phase-adjustments, as is common in shift workers, is associated with early aging phenotypes and reduced lifespan in both mice and flies[35,39]. Interestingly, placing BMAL1 knockout mice on calorie restriction fails to extend their lifespan[40]; although, these mice lack BMAL1 expression in all tissues and throughout development. Our study is the

demonstrates that disruptions to the photoreceptor, and in more specifically disruptions of the CLK function within photoreceptors is sufficient to shorten lifespan.

DR extends lifespan in part by maintaining photoreceptor homeostasis

A number of studies have previously investigated the effects of light exposure on lifespan in *Drosophila*[41]. These studies, however, have not simultaneously examined the influence of diet and the influence of the photoreceptor cells. Exposure to short-wavelength light (i.e., blue-light), which is especially phototoxic, reduces survival in worms and flies[22,42]. Interestingly, housing flies in a 12:12 blue-light/dark cycle significantly shortens lifespan even when those flies lack photoreceptors[22]. This effect appears to be directly related to blue-light mediated neuronal cell death (i.e., extraocular blue light sensing). Our lab previously demonstrated that DR-mediated lifespan extension is completely abolished in flies reared in constant lighting conditions (LL), while flies reared on AL experienced only a minor decrease in lifespan in LL[43]. We previously attested that LL blocked DR's lost ability to extend lifespan because it induces arrhythmicity. An alternative hypothesis is that the LL-mediated lifespan shortening on DR was also a result of the phototoxic effects of LL, which force photoreceptors to rapidly degenerate. The observation that knocking down $ATP\alpha$ in the eye (a model of forced photoreceptor degeneration) also significantly reduced lifespan on DR, further supports the photoreceptor hypothesis. Furthermore, as discussed above, photoreceptors regulate the timing of their light-sensitivity through the molecular clock. Therefore, housing a fly in LL would likely render their photoreceptor clocks arrhythmic, and increase the photoreceptor cells' susceptibility to phototoxic stress. Interestingly, chronic dim-light exposure at night also shortens lifespan in *Drosophila*[44]. Our results here, indicate that DR protects flies from the lifespan-shortening effects of photoreceptor activation.

Although, forced photoreceptor degeneration is sufficient to significantly reduce longevity on DR, we also demonstrate that DR protects against the lifespan-shortening effects of photoreceptor activation during a normal 12:12 LD cycle. Flies reared on AL, which display dampened circadian rhythms within the eye, were selectively sensitive to lethality from the optogenetic activation of the photoreceptors. Inversely, we report that white-eyed flies, which are highly susceptible to light-mediated retinal degeneration, only display lifespan extension from constant darkness when they are reared on AL. Consistent with this observation, Rh null flies (which have reduced photoreceptor activity) display proportionally larger increases in lifespan when maintained on AL vs. DR. Importantly, although Rh expression is enriched in the photoreceptor cells, it is also expressed in other populations of neurons. Therefore, we cannot be certain that the lifespan extensions we observe in Rh null flies is solely the result of diminished Rh levels in the photoreceptor cells.

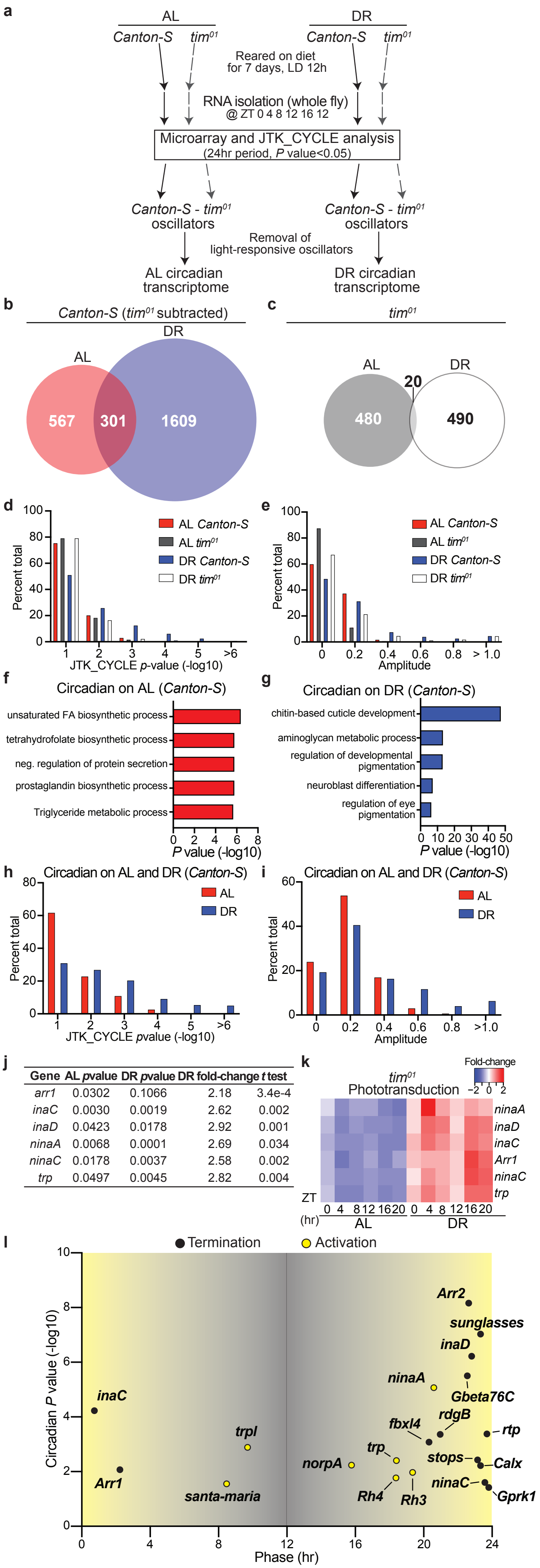

Gene	AL pvalue	DR pvalue	DR fold-change	t test
arr1	0.0302	0.1066	2.18	3.4e-4
inaC	0.0030	0.0019	2.62	0.002
inaD	0.0423	0.0178	2.92	0.001
ninaA	0.0068	0.0001	2.69	0.034
ninaC	0.0178	0.0037	2.58	0.002
trp	0.0497	0.0045	2.82	0.004

Supplementary Figure 1. Dietary restriction amplifies circadian transcriptional output in wild-type, but not *tim⁰¹* circadian mutants. (a) Experimental design of the time-course microarray and identification of circadian transcripts in in *Canton-S* and tim^{01} flies. Females were reared on AL or DR diets for 7 days. Flies were then collected, and mRNA was isolated from whole-fly lysates at 4-hour intervals for 24 hours (*n*=4 pooled mRNA samples from 30 flies per condition/timepoint). Circadian transcripts were identified with the JTK_CYCLE algorithm[45]. Oscillatory transcripts in tim^{01} flies were subtracted from oscillating transcripts in wildtype flies for each diet to control for the influence of rhythmic lighting conditions. (b-c) Venn-diagrams displaying the number of circadian transcripts that oscillate in flies reared on AL, DR, or in both diets for *Canton-S* (b) or tim^{01} mutants (c). (d-e) Histograms of JTK_CYCLE *P* value (non-adjusted) statistics (d) and circadian amplitudes (f) of transcripts that cycle on AL or DR diets in *Canton-S* and tim^{01} flies. The *y*-axis indicates the precent of transcripts that oscillate on each diet. (f-g) Gene-ontology enrichment categories corresponding to transcripts that cycle (JTK_CYCLE $P \leq 0.05$, non-adjusted) on AL (f) or DR (g) in *Canton-S* flies. (h-i) Histograms of JTK_CYCLE *P* value (non-adjusted) statistics (h) and circadian amplitudes (i) of transcripts that are circadian on both diets in *Canton-S* flies. The *y*-axis indicates percent of transcripts out of the 301 total transcripts that oscillate on both diets. (j) Table of phototransduction genes that are circadian on AL and DR. **cry* is only circadian in DR, and *arr1* is only circadian on DR. Fold-changes and Student's *t* test (two-sided, paired) statistics were calculated by averaging the individual fold-changes in expression for each timepoint. Circadian *P* values were calculated by the JTK_CYCLE algorithm (non-adjusted). (k) Heatmap of phototransduction transcript expression on AL and DR in tim^{01} flies. (l) Circadian acrophase diagram of phototransduction/light-response transcripts

reported in Kuintzle *et al*., 2017[7] plotted per their phase (time of peak expression). *P* values for circadian rhythmicity were calculated with ARSER.

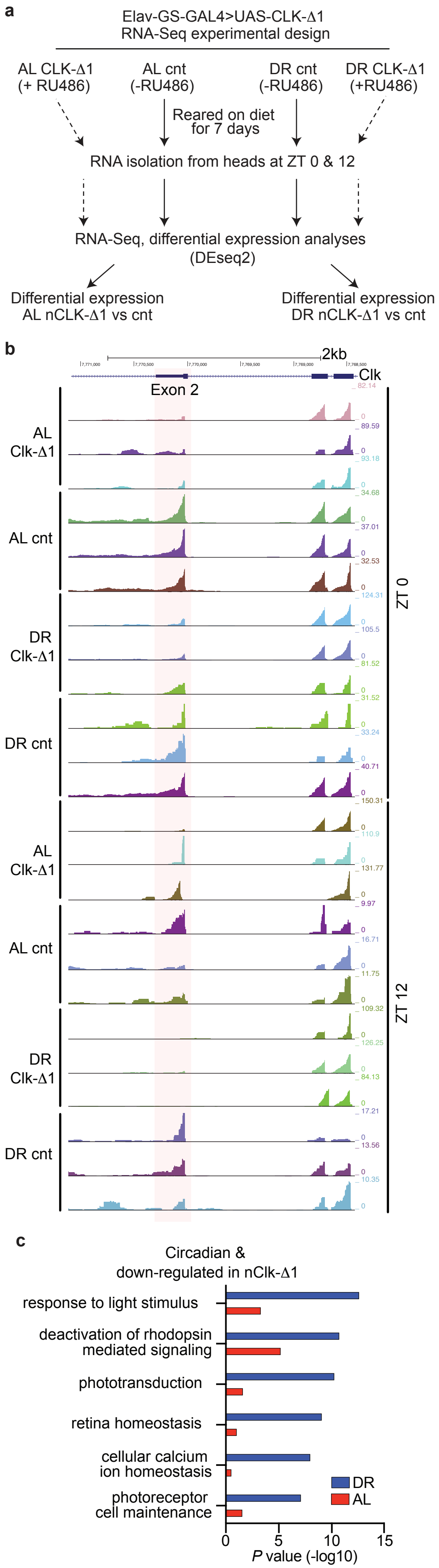
a
Elav-GS-GAL4>UAS-CLK-Δ1
RNA-Seq experimental design
AL CLK-Δ1
(+ RU486)
AL cnt
(-RU486)
DR cnt
(-RU486)
DR CLK-Δ1
(+RU486)
Reared on diet
for 7 days
RNA isolation from heads at ZT 0 & 12
RNA-Seq, differential expression analyses
(DEseq2)
Differential expression
AL nCLK-Δ1 vs cnt
Differential expression
DR nCLK-Δ1 vs cnt
b
2kb
Clk
Exon 2
AL
Clk-Δ1
AL cnt
DR
Clk-Δ1
DR cnt
ZT 0
ZT 12
c
Circadian &
down-regulated in nClk-Δ1
response to light stimulus
deactivation of rhodopsin
mediated signaling
phototransduction
retina homeostasis
cellular calcium
ion homeostasis
photoreceptor
cell maintenance
DR
AL
0
5
10
15
P value (-log10)

Supplementary Figure 2. Design and additional analyses of nCLK-Δ1 RNA-Seq. (a) Design of nCLK-Δ1 RNA-Seq. Mated females were reared on AL or DR with the addition of vehicle or RU486 to induce the expression of CLK-Δ1 pan-neuronally for 7 days. mRNA was isolated from heads (*n*=3 biological replicates, *N*=30 heads per replicates) at ZT0 and ZT12. RNA-sequencing was performed and differentially expressed genes were identified with the DEseq2 software[46] package. (b) UCSC genome browser visualization of the individual tracks for each nCLK-Δ1 RNA-Seq sample zoomed into exon 2 of *clk* (chr3L:7,766,807-7,773,169). Exon 2 (highlighted in red) of *clk* encodes the basic helix-loop-helix domain (DNA binding) of CLK that is selectively ablated in CLK-Δ1 flies. Overexpression of CLK-Δ1 results in a relative decrease in the ratio of tags at exon 2 vs exon 3-4 (right), while exon 3-4 display elevated tag density compared to control samples. Track size is normalized for each sample and the total number of tags is indicated as the top number (color coded to match each track) on the far right. (c) Gene-ontology enrichment terms and *P* value statistics for genes that are circadian in young heads (ARSER $P \leq 0.05$, GEO81100)[7] and significantly down-regulated in the nCLKΔ1 RNA-Seq on AL or DR. *P* values were calculated with hypergeometric distribution (findGO.pl, HOMER) with no adjustment for multiple-hypothesis testing.

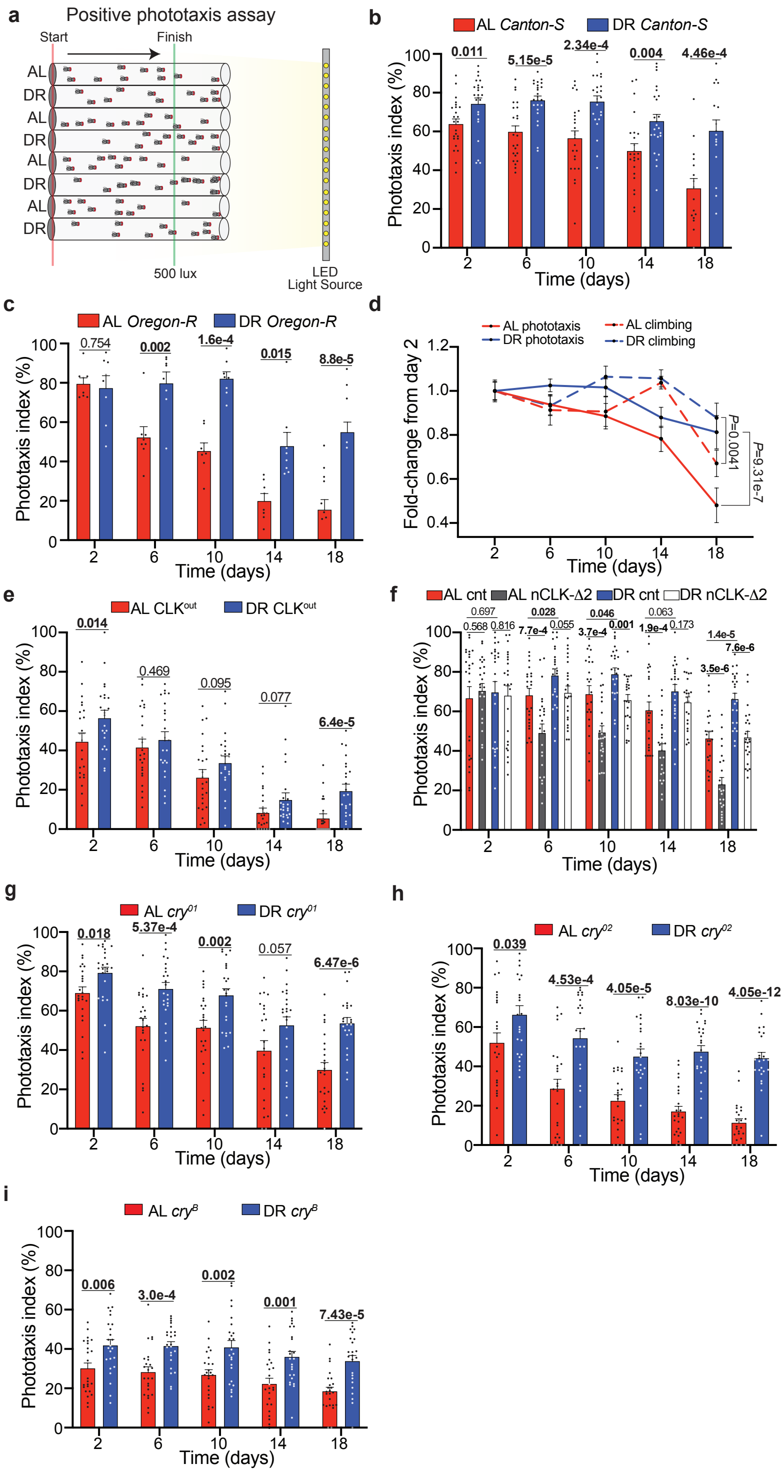
a
Positive phototaxis assay
Start
Finish
AL
DR
500 lux
LED
Light Source
b
AL Canton-S
DR Canton-S
Phototaxis index (%)
Time (days)
c
AL Oregon-R
DR Oregon-R
d
AL phototaxis
DR phototaxis
AL climbing
DR climbing
Fold-change from day 2
P=0.0041
P=9.31e-7
e
AL CLKout
DR CLKout
f
AL cnt
AL nCLK-Δ2
DR cnt
DR nCLK-Δ2
g
AL cry01
DR cry01
h
AL cry02
DR cry02
i
AL cryB
DR cryB

Supplementary Figure 3. Design and additional analyses of positive phototaxis assays in wild-type and circadian mutants. (a) Diagram of positive-phototaxis setup. Flies are sorted in clear elongated fly vials, dark adapted for 15 minutes, knocked to the bottom of the vial, and then laid horizontally and perpendicular to an LED light source. Once the light is turned on flies that reach the green line are scored as "positive-phototaxis" and counted at 15, 30, 45 seconds (See methods for additional details). (b) Positive phototaxis responses for *Canton-S* females reared on AL or DR diets. See methods for calculation of phototaxis index. For each timepoint results are represented as average percent positive phototaxis ±SEM (*n*=24 biological reps, *N*=480 flies per condition). (c) Phototaxis responses for *Oregon-R* females. For each timepoint results are represented as average percent phototaxis response ±SEM (*n*=8 biological replicates, *N*=160 flies per condition). (d) *Canton-S* climbing activity and positive phototaxis plotted as fold-change from responses at day 2. Data are presented as mean values and the error bars indicate ±SEM (*n*=24 biologically independent cohorts of 20 flies examined over three independent experiments, *N*=480 flies per condition). *P* values were calculated with a mixed-effect ANOVA comparing the values for climbing and phototaxis between flies reared on AL and DR. (e) Positive phototaxis responses for Clk^{out} females reared on AL or DR diets. For each timepoint results are represented as average percent positive phototaxis ±SEM (*n*=24 biological reps, *N*=480 flies per condition). (f) Positive phototaxis responses for nCLK-Δ2 flies (Elav-GS-GAL4>UAS-CLK-Δ2. For each timepoint results are represented as average percent positive phototaxis ±SEM (*n*=24 biological replicates, *N*=480 flies per condition). (g-i) Positive phototaxis responses for cry^{01} (g), cry^{02} (h), cry^{B} (i) females reared on AL or DR diets. For each timepoint results are represented as average percent positive phototaxis ±SEM (*n*=24 biological reps, *N*=480 flies per condition). (b-c and e-i) *P* values

were determined by two-tailed Student's *t* test (unpaired) at each timepoint. Source data are provided as a Source Data file.

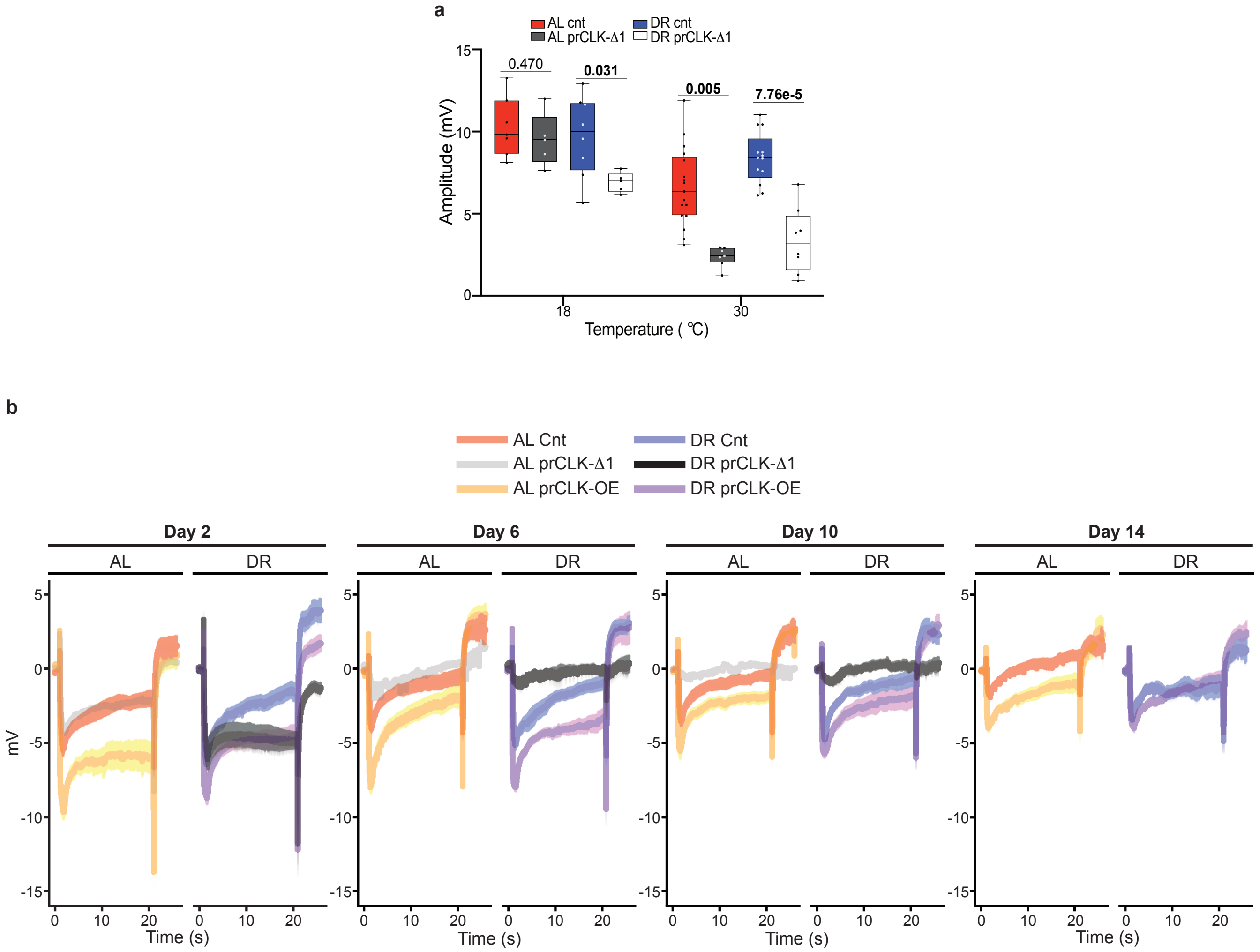

a
AL cnt
AL prCLK-Δ1
DR cnt
DR prCLK-Δ1
0.470
0.031
0.005
7.76e-5
Amplitude (mV)
Temperature (℃)
b
AL Cnt
AL prCLK-Δ1
AL prCLK-OE
DR Cnt
DR prCLK-Δ1
DR prCLK-OE
Day 2
Day 6
Day 10
Day 14
AL
DR
mV
Time (s)

Supplementary Figure 4. Electroretinogram analyses and representative traces of photoreceptor-specific modulation of CLK. (a) Box-plots of electroretinogram amplitudes for prCLK-Δ1 (Trpl-GAL4;GAL80>UAS-CLK-Δ1^{OC}) and control flies (Trpl-GAL4;GAL80>*Canton-S*^{OC}) reared at 18$^{\circ C}$ (GAL80 active, GAL4 inactive) and 30$^{\circ C}$ (GAL80 inactive, GAL4 active) for 6 days. Illuminance was set at 15000 Lux. Data are presented as Tukey multiple comparison of means: The horizontal line within each box is the median, the bottom and top of the box are lower and upper quartiles, and the whiskers are minimum and maximum values. The number of biologically independent flies measured at each condition are as follows at 18$^{\circ C}$ and 30$^{\circ C}$, respectively: AL cnt 7, 17 flies, AL prCLK-Δ1 5, 7 flies, DR cnt 8, 13 flies, DR prCLK-Δ1 5, 8 flies. The data were collected over 1 independent experiment. *P* values were determined by two-tailed Student's *t* test (unpaired), comparing responses between genotypes. (b) Average ERG traces from day 2 to 14 in prCLK-Δ1, prCLK-OE (Trpl-GAL4;GAL80>UAS-*Clk*), and control flies reared at 30$^{\circ C}$. Source data are provided as a Source Data file.

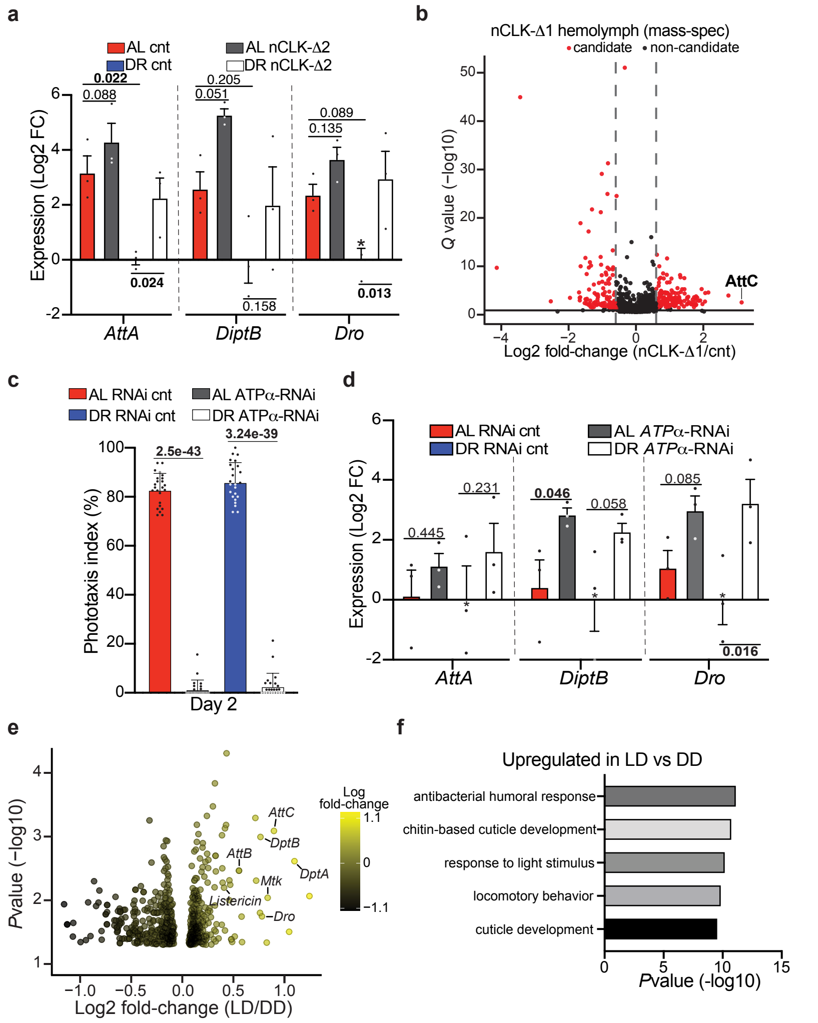
a
AL cnt
DR cnt
AL nCLK-Δ2
DR nCLK-Δ2
Expression (Log2 FC)
0.022
0.088
0.024
0.205
0.051
0.158
0.089
0.135
0.013
AttA
DiptB
Dro
b
nCLK-Δ1 hemolymph (mass-spec)
candidate
non-candidate
Q value (−log10)
AttC
Log2 fold-change (nCLK-Δ1/cnt)
c
AL RNAi cnt
DR RNAi cnt
AL ATPα-RNAi
DR ATPα-RNAi
2.5e-43
3.24e-39
Phototaxis index (%)
Day 2
d
AL RNAi cnt
DR RNAi cnt
AL ATPα-RNAi
DR ATPα-RNAi
Expression (Log2 FC)
0.445
0.231
0.046
0.058
0.085
0.016
AttA
DiptB
Dro
e
Pvalue (−log10)
Log fold-change
AttC
DptB
AttB
DptA
Mtk
Listericin
Dro
Log2 fold-change (LD/DD)
f
Upregulated in LD vs DD
antibacterial humoral response
chitin-based cuticle development
response to light stimulus
locomotory behavior
cuticle development
Pvalue (-log10)

Supplementary Figure 5. Immune responses in nCLK, *ATPalpha* knockdown, and wild-type flies reared in different lighting conditions. (a) Relative expression of AMP genes (*AttA*, *DiptB*, and *Dro*) calculated by RT-qPCR with mRNA isolated from nCLK-Δ2 bodies. Results are plotted as average Log2 fold-change in expression calculated by the ΔΔ-Ct method, normalized to DR vehicle treated control samples as well as *rp49* ±SEM (*n*=3 biological replicates, *N*=30 flies per biological replicate). (b) Volcano-plot of hemolymph proteins identified by tandem mass-spectrometry comparing nCLK-Δ1 (RU486 treated, *N*=300) and control (vehicle treated, *N*=300) flies reared on AL at day 14. Each dot represents an individual protein with a statistical significance $Q \leq 0.0001$ comparing nCLK-Δ1 and control hemolymph samples. Red dots are differentially expressed protein candidates with a Log2 fold-change cutoff of ± 0.6. A description of the statistical analysis is in the Statistics and Reproducibility section. (c) Positive phototaxis responses with eye-specific knockdown of $ATP\alpha$ (GMR-GAL4>UAS-$ATP\alpha$-RNAi) compared to RNAi control flies (GMR-GAL4>UAS-mCherry-RNAi). For each timepoint results are represented as average phototaxis response ±SEM (*n*=24 biological replicates, *N*=480 flies per condition). *P* values were determined by two-tailed Student's *t* test (unpaired) at each timepoint. (d) Relative mRNA expression of AMP genes calculated by RT-qPCR with mRNA isolated from bodies of eye-specific ATPalpha knockdown flies (GMR-GAL4>UAS-ATPalpha-RNAi) vs RNAi control flies (GMR-GAL4>UAS-mCherry-RNAi). Results are plotted as average Log2 fold-change in expression calculated by the DD-Ct method, normalized to DR RNAi control samples as well as housekeeping gene rp49±SEM (*n*=3 biological replicates, N=30 flies per biological replicate). *P* values were calculated with the two-sided Student's *t* test (unpaired) comparing Log2 fold-changes in expression. (e) Volcano-plot of gene expression changes in heads of *y.w.* flies housed

in 12:12 LD vs constant darkness (DD) from Wijnen *et al*., 2006 (GSE3842)[47]. *P* values were determined by two-tailed Student's *t* test (paired), comparing expression changes across all time-points. (f) The top-5 enriched gene-ontology categories corresponding to genes that are upregulated in heads of flies housed in LD vs DD. *P* values were calculated with hypergeometric distribution (findGO.pl, HOMER) with no adjustment for multiple-hypothesis testing. Source data are provided as a Source Data file.

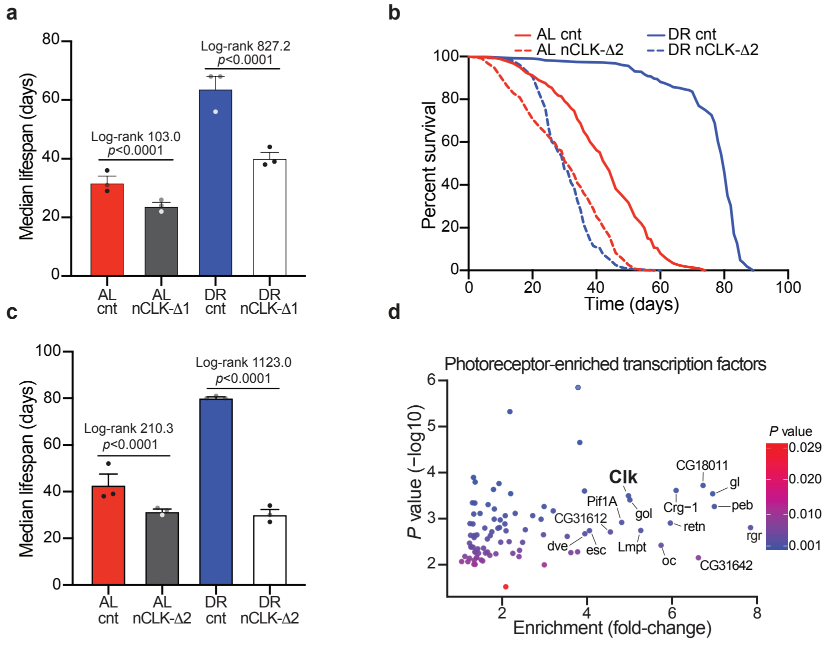

a
Log-rank 103.0
p<0.0001
Log-rank 827.2
p<0.0001
Median lifespan (days)
AL cnt
AL nCLK-Δ1
DR cnt
DR nCLK-Δ1
b
AL cnt
AL nCLK-Δ2
DR cnt
DR nCLK-Δ2
Percent survival
Time (days)
c
Log-rank 210.3
p<0.0001
Log-rank 1123.0
p<0.0001
Median lifespan (days)
AL cnt
AL nCLK-Δ2
DR cnt
DR nCLK-Δ2
d
Photoreceptor-enriched transcription factors
P value (−log10)
Enrichment (fold-change)
P value
Clk
CG18011
gl
peb
Crg−1
retn
rgr
Pif1A
gol
CG31612
dve
esc
Lmpt
oc
CG31642

Supplementary Figure 6. Median lifespan of nCLK-Δ flies and photoreceptor enriched transcription factors. (a) Median lifespan of nCLK-Δ1 flies corresponding to lifespans in (Fig. 4e). Data are plotted as the average median lifespan of the 3 biological replicates and error bars indicate ±SEM. *P* values were determined by Chi square from Log-rank (Mantel-Cox) test. $P<1.0e-15$ for both AL and DR. (b) Survival analysis of nCLK-Δ2 flies. Survival data is plotted as an average of three independent lifespan repeats. Control flies (vehicle treated): AL $N=505$, DR $N=504$; nCLK-Δ1 flies (RU486 treated): AL $N=497$, DR $N=508$. (c) Median lifespan of nCLK-Δ2 flies. Data are plotted as the average median lifespan of the 3 biological replicates and error bars indicate ±SEM. *P* values were determined by Chi square from Log-rank (Mantel-Cox) test. $P\leq1.0e-15$ for both AL and DR. (d) Volcano-plot of photoreceptor-enriched transcription factors from Hall *et al.*, 2017 (GSE93128)[48]. *P* values were determined by two-tailed Student's *t* test (unpaired), comparing expression changes from pre- and post-enrichment. Source data are provided as a Source Data file.

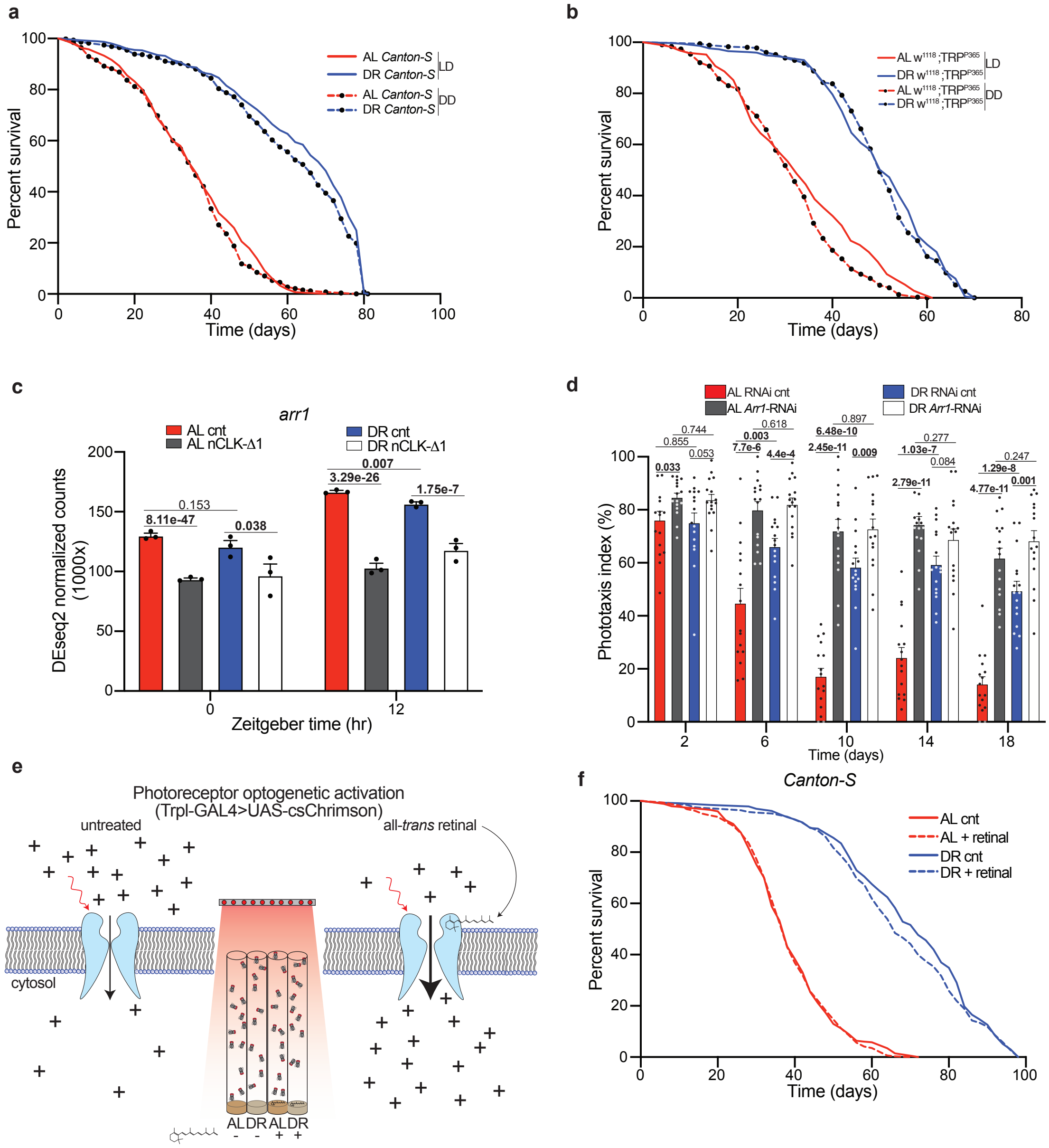

a
Percent survival
Time (days)
AL Canton-S
DR Canton-S
LD
AL Canton-S
DR Canton-S
DD
b
Percent survival
Time (days)
AL w1118;TRPP365
DR w1118;TRPP365
LD
AL w1118;TRPP365
DR w1118;TRPP365
DD
c
arr1
AL cnt
AL nCLK-Δ1
DR cnt
DR nCLK-Δ1
DEseq2 normalized counts (1000x)
Zeitgeber time (hr)
0.153
8.11e-47
0.038
0.007
3.29e-26
1.75e-7
d
AL RNAi cnt
AL Arr1-RNAi
DR RNAi cnt
DR Arr1-RNAi
Phototaxis index (%)
Time (days)
0.744
0.855
0.033
0.053
0.618
0.003
7.7e-6
4.4e-4
0.897
6.48e-10
2.45e-11
0.009
0.277
1.03e-7
0.084
2.79e-11
0.247
1.29e-8
4.77e-11
0.001
e
Photoreceptor optogenetic activation
(Trpl-GAL4>UAS-csChrimson)
untreated
all-trans retinal
cytosol
AL DR AL DR
f
Canton-S
AL cnt
AL + retinal
DR cnt
DR + retinal
Percent survival
Time (days)

Supplementary Figure 7. Lighting control lifespans, *arr1*-RNAi phototaxis, and optogenetic activation diagram and retinal control lifespans. (a) Survival analysis of *Canton-S* wildtype flies housed in 12:12h LD and constant darkness (DD). Survival data is plotted as an average of three independent lifespan crosses. AL LD *N*=549, AL DD *N*=510, DR LD *N*=558, DR DD *N*=509. (b) Survival analysis of white-eyed, photoreceptor null flies (w^{1118}; TRP^{P365}) housed in 12:12h LD or DD. Survival data is plotted as an average of two independent lifespan repeats. LD housed flies: AL *N*=290, DR *N*=373; DD housed flies: AL *N*=301, DR *N*=357. (c) Normalized expression counts for *arr1* from the nCLK-Δ1 RNA-Seq. Results are represented as average expression counts calculated by DEseq2 ±SEM. *P* values (non-adjusted) were determined by DEseq2 differential expression analysis. *n*=3 biologically independent cohorts of 100 female fly heads per group examined over one independent experiment. (d) Positive phototaxis responses with eye-specific knockdown *arr1* (e, GMR-GAL4>UAS-*arr1*-RNAi compared to RNAi control flies (GMR-GAL4>empty vector, VDRC). For each timepoint results are represented as average phototaxis response ±SEM (RNAi control and *arr1*: *n*=16 biological replicates, *N*=320 flies per condition). *P* values were determined by two-tailed Student's *t* test (unpaired) at each timepoint comparing the phototaxis index of RNAi control flies to *arr1*-RNAi flies. (e) Diagram of optogenetic activation of photoreceptors. The photoreceptor-specific driver, Trpl-GAL4, drives the expression of a red-shifted csChrimson channel in R1-R8 photoreceptors. Addition of all-*trans* retinal (50μM) in the fly media promotes the opening of optogenetic channels in the presence of red-light, allowing the flow of positively charged ions into the cytosol to activate photoreceptors. (f) Survival analysis of *Canton-S* flies reared in 12:12 red-light:dark on AL and DR diets with the addition of all-*trans* retinal or vehicle (control). Survival data is plotted as an average of two

independent lifespan repeats. All-*trans* retinal treated flies: AL *N*= 340, DR *N*=328; Vehicle treated flies: AL *N*=347, DR *N*=328. Source data are provided as a Source Data file.

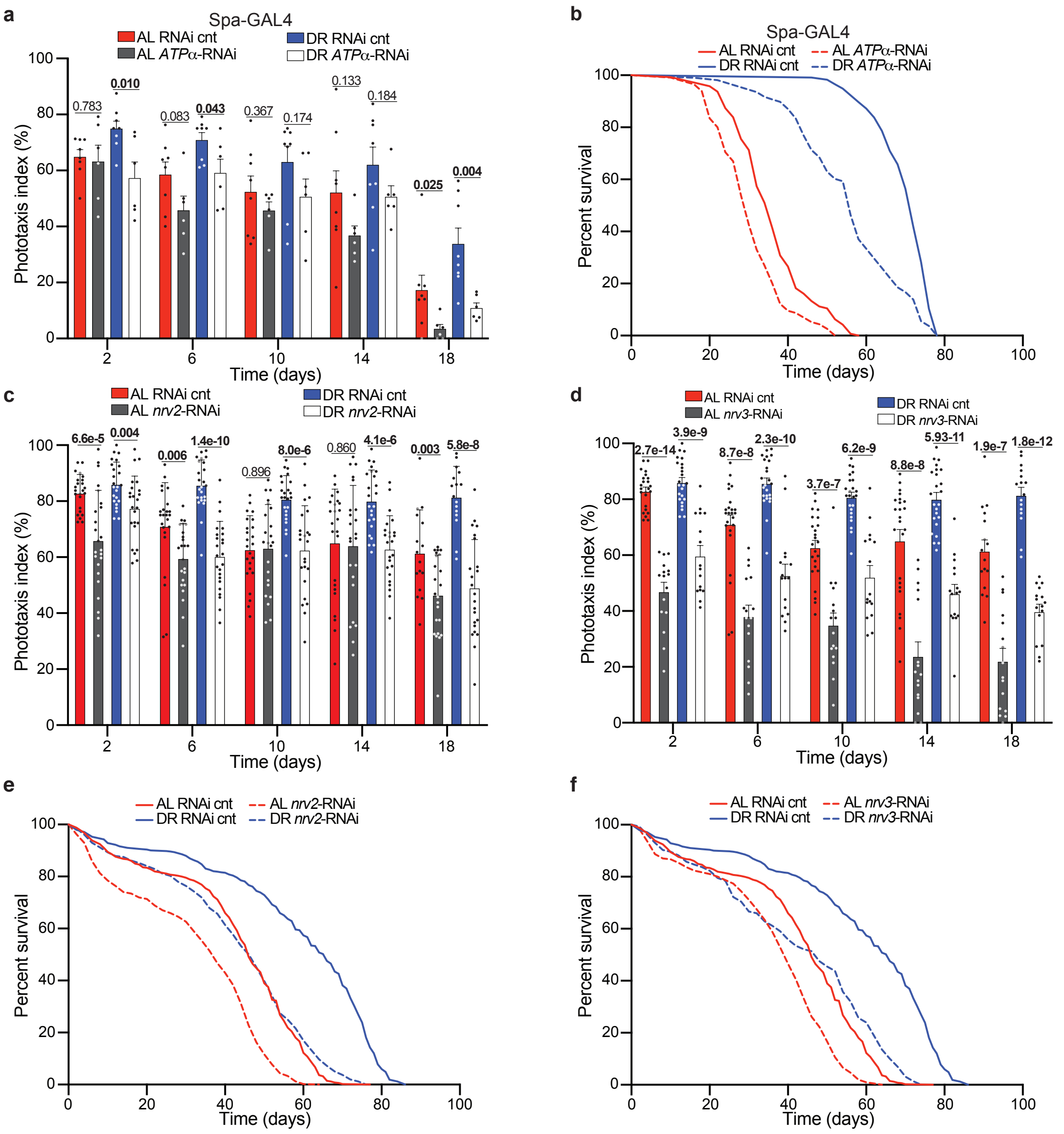
a
Spa-GAL4
AL RNAi cnt
AL ATPα-RNAi
DR RNAi cnt
DR ATPα-RNAi
Phototaxis index (%)
Time (days)
b
Spa-GAL4
AL RNAi cnt
DR RNAi cnt
AL ATPα-RNAi
DR ATPα-RNAi
Percent survival
Time (days)
c
AL RNAi cnt
AL nrv2-RNAi
DR RNAi cnt
DR nrv2-RNAi
Phototaxis index (%)
Time (days)
d
AL RNAi cnt
AL nrv3-RNAi
DR RNAi cnt
DR nrv3-RNAi
Phototaxis index (%)
Time (days)
e
AL RNAi cnt
DR RNAi cnt
AL nrv2-RNAi
DR nrv2-RNAi
Percent survival
Time (days)
f
AL RNAi cnt
DR RNAi cnt
AL nrv3-RNAi
DR nrv3-RNAi
Percent survival
Time (days)

Supplementary Figure 8. Positive phototaxis and lifespan analyses of cone-cell specific knockdown of *ATPα* and eye-specific knockdown of *ATPα*-subunits. (a) Positive phototaxis responses with cone-cell specific knockdown *ATPα* (Spa-GAL4>UAS-*ATPα*-RNAi) compared to RNAi control flies (GMR-GAL4>UAS-mCherry-RNAi). For each timepoint results are represented as average phototaxis response ±SEM (RNAi control and *nrv2*: *n*=8 biological replicates, *N*=140 flies per condition). (b) Survival analysis of cone-cell specific knockdown of *ATPα* compared to RNAi control flies. Survival data is plotted as from 1 independent lifespan repeat for RNAi controls and *ATPα* RNAi flies. RNAi cnt flies: AL *N*=143, DR *N*=117; *ATPα* RNAi flies: AL *N*=115, DR *N*=108. (c-d) Positive phototaxis responses with eye-specific knockdown *nrv2* (c, GMR-GAL4>UAS-*nrv2*-RNAi), and *nrv3* (d, GMR-GAL4>UAS-*nrv3*-RNAi) compared to RNAi control flies (GMR-GAL4>UAS-*mCherry*-RNAi). For each timepoint results are represented as average phototaxis response ±SEM (RNAi control and *nrv2*: *n*=24 biological replicates, *N*=480 flies per condition; *nrv3*: *n*=16 biological replicates, *N*=320 flies per condition). (e-f) Survival analysis of eye-specific *nrv2* (e) and *nrv3* (f) RNAi knockdown flies compared to RNAi control flies. Survival data is plotted as an average of three independent lifespan repeats for RNAi controls and *nrv2* RNAi flies, and two independent lifespan crosses for *nrv3* RNAi knockdown flies. RNAi cnt flies: AL *N*=493, DR *N*=490; *nrv2* RNAi flies: AL *N*=482, DR *N*=513; *nrv3* RNAi flies: AL *N*=301, DR *N*=288. (a, c-d) *P* values were determined by two-tailed Student's *t* test (unpaired) at each timepoint. Source data are provided as a Source Data file.

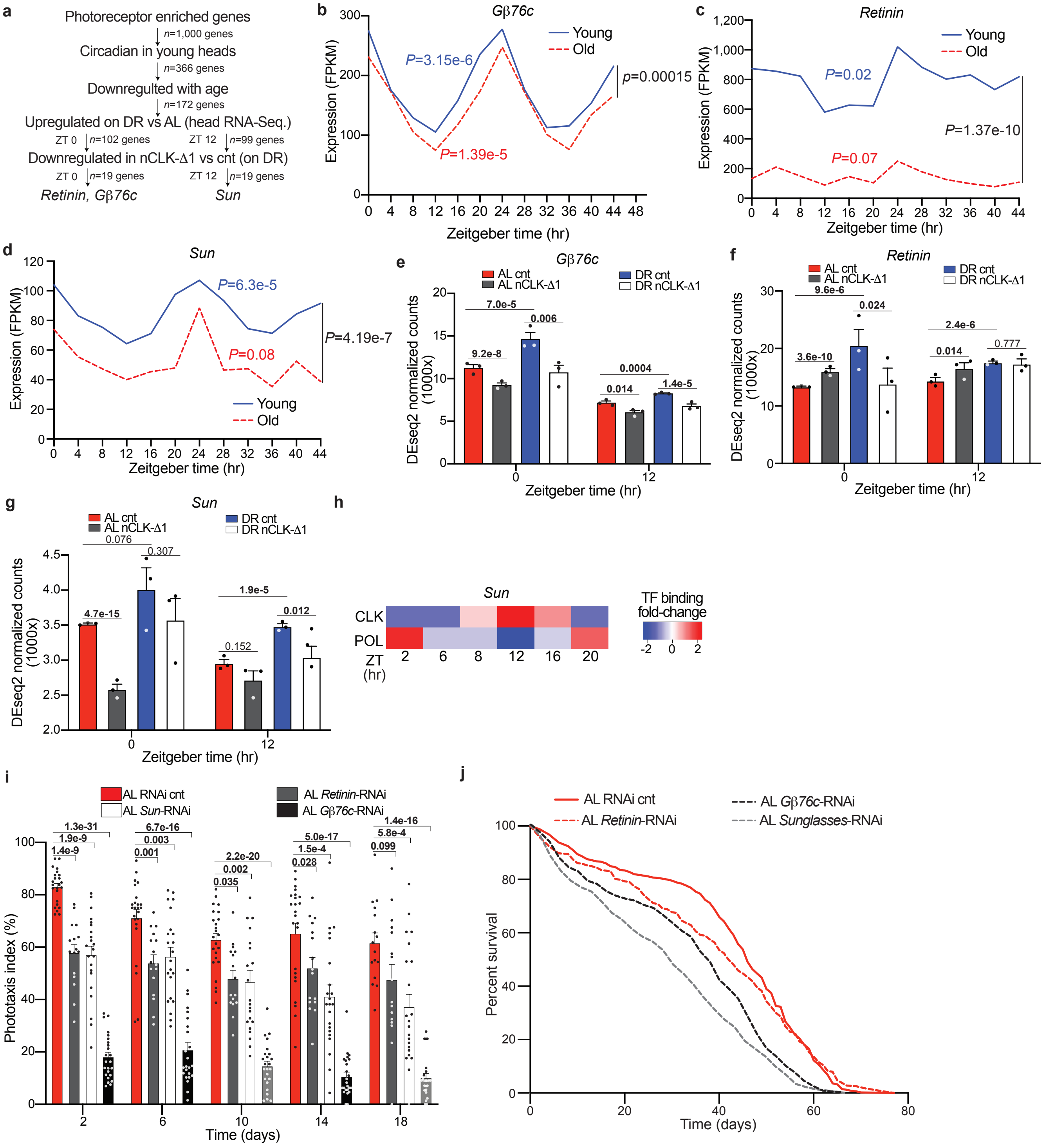

a
Photoreceptor enriched genes
n=1,000 genes
Circadian in young heads
n=366 genes
Downregulted with age
n=172 genes
Upregulated on DR vs AL (head RNA-Seq.)
ZT 0 n=102 genes
ZT 12 n=99 genes
Downregulated in nCLK-Δ1 vs cnt (on DR)
ZT 0 n=19 genes
ZT 12 n=19 genes
Retinin, Gβ76c
Sun
b
Gβ76c
Young
Old
P=3.15e-6
P=1.39e-5
p=0.00015
Expression (FPKM)
Zeitgeber time (hr)
c
Retinin
P=0.02
P=0.07
P=1.37e-10
d
Sun
P=6.3e-5
P=0.08
P=4.19e-7
e
Gβ76c
AL cnt
AL nCLK-Δ1
DR cnt
DR nCLK-Δ1
DEseq2 normalized counts (1000x)
7.0e-5
0.006
9.2e-8
0.0004
0.014
1.4e-5
f
Retinin
9.6e-6
0.024
3.6e-10
2.4e-6
0.014
0.777
g
Sun
0.076
0.307
4.7e-15
1.9e-5
0.012
0.152
h
Sun
CLK
POL
ZT (hr)
2 6 8 12 16 20
TF binding fold-change
-2 0 2
i
AL RNAi cnt
AL Sun-RNAi
AL Retinin-RNAi
AL Gβ76c-RNAi
Phototaxis index (%)
Time (days)
1.3e-31
1.9e-9
1.4e-9
6.7e-16
0.003
0.001
2.2e-20
0.002
0.035
5.0e-17
1.5e-4
0.028
1.4e-16
5.8e-4
0.099
j
AL RNAi cnt
AL Retinin-RNAi
AL Gβ76c-RNAi
AL Sunglasses-RNAi
Percent survival
Time (days)

Supplementary Figure 9. Identification of photoreceptor enriched CLK-output genes, and additional analyses with eye-specific knockdown of *Gβ76c*, *retinin*, and *sun*. (a) Bioinformatics pipeline for identifying photoreceptor enriched, CLK-output genes. (b-d) Circadian expression of *Gβ76c*, *retinin*, and *sun* and their corresponding circadian *P* value statistics for young (5-day old) and old (55-day old) wildtype heads from Kuintzle *et al*., 2017[7]. Circadian *P* values were determined by ARSER algorithm by Kuintzle *et al*., 2017[7] (AL=red, DR=blue). To compare gene expression profiles with age we utilized the two-tailed Student's *t test* (paired) to determine *P* values (black). (e-g) Normalized expression counts for *Gβ76c*, *retinin*, and *sun* from the nCLK-Δ1 RNA-Seq. Results are represented as average expression counts calculated by DEseq2 ±SEM. *P* values (non-adjusted) were determined by DEseq2 differential expression analysis. *n*=3 biologically independent cohorts of 100 female fly heads per group examined over one independent experiment. (h) Heatmap of CLK and POL (*Drosophila* polymerase) tag-densities at the 5′-untranslated region of the *sun* promoter over a circadian time-course from ChIP-Chip analyses[49]. Consistent with other direct CLK target genes, Abruzzi *et al*., 2011 report maximal CLK binding at ZT 12, while POL displayed antiphasic binding to that of CLK and aligned with the phase of *sun* mRNA expression (ZT 0-2). *CLK binding was not observed in GMR-HID heads suggesting *sun* is under CLK transcriptional regulation specifically in the neurons of the eye. (i) Positive phototaxis responses with eye-specific knockdown of *Gβ76c* (GMR-GAL4>UAS- *Gβ76c*-RNAi), *retinin* (GMR-GAL4>UAS-*retinin*-RNAi), and *sun* (GMR-GAL4>UAS-*sun*-RNAi) compared to RNAi control flies (GMR-GAL4>UAS-mCherry-RNAi) reared on AL. For each timepoint results are represented as average phototaxis response ±SEM (RNAi control *n*=24 biological replicates, *N*=480 flies per condition; *Gβ76c* RNAi *n*=24 biological replicates, *N*=480 flies per condition, *retinin* RNAi

n=16 biological replicates, *N*=384 flies per condition; *sun* RNAi *n*=24 biological replicates, *N*=480 flies per condition). *P* values were determined by two-tailed Student's *t* test (unpaired) at each timepoint comparing the phototaxis index of RNAi control flies to *retinin*- and *sun*-RNAi flies. (j) Survival analysis of eye-specific *Gβ76c*-RNAi, *retinin*-RNAi, *sun*-RNAi, and RNAi control knockdown flies compared to RNAi control flies reared on AL. Survival data is plotted as an average of three independent lifespan repeats for RNAi control, *Gβ76c*-RNAi, *sun*-RNAi flies and two independent lifespan repeats for *retinin*-RNAi flies. RNAi cnt flies: *N*=493; *Gβ76c* RNAi flies: *N*=543; *retinin* RNAi flies: *N*=353; *sun* RNAi flies: *N*=503. Source data are provided as a Source Data file.

Gene symbol	Gene name	young *P*value	old *P*value	AL *P*value	DR *P*value	CLK binding
Arr1	Arrestin 1	0.0084	0.0040	0.0302	0.1066	* †
Arr2	Arrestin 2	6.89e-09	0.0096	1	1	
Calx	Na/Ca-exchange protein	0.0059	0.0001	1	0.1623	* †
Camta	Calmodulin-binding transcription factor	0.0158	0.0029	1	1	*
CdsA	CDP diglyceride synthetase	8.67e-06	0.0026	1	0.1856	* †
Cib2	Calcium and integrin binding family member 2	0.4123	0.0301	1	0.7501	
cl	clot	0.0069	0.0406	1	1	* †
cry	cryptochrome	0.0006	0.0005	0.1415	4.07e-7	*
Dmn	Dynactin 2, p50 subunit	0.0084	0.0401	1	1	
Ekar	Eye-enriched kainate receptor	0.0170	0.0432	1	0.6100	
Fbxl4	F box and leucine-rich-repeat gene 4	0.0008	0.5073	0.2729	0.0583	* †
Galphaq	G protein alpha q subunit	0.1135	0.0076	1	1	* †
Gbeta76c	G protein beta-subunit 76C	3.15e-06	1.39e-05	1	1	
Gprk1	G protein-coupled receptor kinase 1	0.0378	0.0260	1	1	*
Gycalpha99B	Guanylyl cyclase alpha-subunit at 99B	0.0060	0.0208	1	1	
Ih	I[[h]] channel	0.0001	0.0002	1	0.3086	* †
inaC	inactivation no afterpotential C	5.92e-05	0.0001	0.0030	0.0012	
inaD	inactivation no afterpoteinal D	5.99e-07	2.48e-05	0.0423	0.0178	
Inx3	Innexin 3	0.0498	0.0212	1	0.0213	
Inx7	Innexin 7	0.0254	0.3187	1	0.0583	
Itpr	Inositol 1,4,5,-tris-phosphate receptor	0.0261	0.1080	1	1	
laza	lazaro	0.0049	0.0024	1	0.0148	
Lrpprc	Leucine-rich pentatricopeptide repeat containing 2	0.0136	0.0918	0.8288	0.3086	
ninaA	neither inactivation nor afterpotential A	8.46e-06	0.0025	0.0068	0.0001	
ninaB	neither inactivation nor afterpotential B	0.3094	0.0049	1	0.9134	
ninaC	neither inactivation nor afterpotential C	0.0251	0.0001	0.0178	0.0037	* †
ninaD	neither inactivation nor afterpotential D	0.0241	0.1043	1	1	
ninaE	neither inactivation nor afterpotential E	0.1575	0.0044	0.3480	1	* †
norpA	no receptor potential A	0.0057	0.0447	1	0.0024	* †
PAPLA1	Phosphatidic Acid Phospholipase A1	0.0241	0.8591	1	1	*
Pdh	Photoreceptor dehydrogenase	0.0017	0.0044	0.0003	3.37e-05	
pinta	prolonged depolarization afterpotential (PDA) is not apparent	0.0346	0.3933	1	1	
PIP5k59B	Phosphatidylinositol 4-phosphate 5-kinase 59B	0.2862	0.0159	1	0.2729	
Pld	Phospholipase D	0.0290	0.0860	1	1	* †
porin	porin	0.0095	0.0017	0.8288	1	*
rdgB	retinal degeneration B	0.0004	0.3399	1	1	* †
rdgC	retinal degeneration C	0.0681	0.0766	1	0.3914	* †
rdhB	retinol dehydrogenase B	2.70e-05	0.0074	0.4911	0.0148	
Rh3	Rhodopsin 3	0.0107	0.4194	1	1	
Rh4	Rhodopsin 4	0.0169	0.2723	1	1	
Rh5	Rhodopsin 5	0.0546	0.0029	0.0068	0.0030	*
Rh6	Rhodopsin 6	0.1415	0.0097	0.0030	0.0148	
rtp	retinophilin	0.0004	0.0272	1	1	
santa-maria	scavenger receptor acting in neural tissue and majority of rh is absent	0.0284	0.0002	1	0.0123	
shakB	shaking B	0.0120	0.0304	0.0084	0.5481	* †
stmA	stambha A	0.0074	0.0045	1	0.4390	* †
stops	slow termination of phototransduction	0.0037	0.0008	1	1	* †
trp	transient receptor potential	0.0040	0.0121	0.0497	0.0045	* †
TrpA1	Transient receptor potential cation channel A1	0.1622	0.0208	0.6773	0.3914	
trpl	transient receptor potential-like	0.0013	0.0002	0.0084	0.0009	
Tsp42Ej/sun	Tetraspanin 42Ej (sunglasses)	6.29e-05	0.0774	1	1	* †
Xport-A	exit protein of rhodopsin and TRP A	0.0022	0.0002	1	1	

* Clk binding
† CLK binding is eye-specific

Supplementary Table 1. Circadian statistics and CLK binding of light-response genes. Circadian *P* value (non-adjusted) statistics for light response genes in young and old wildtype heads (calculated by ARSER by Kuintzle et al., 2017 [7]) and whole flies reared on AL and DR (calculated by JTK_CYCLE in this study).

Drosophila strain	Genotype	Source	Catalog #
w^{1118}	w^{1118}	This manuscript	N/A
$ninaE^{17}$ outcrossed to w^{1118}	w^{1118}; $ninaE^{17}$	This manuscript	N/A
$rh3^{2}$ outcrossed to w^{1118}	w^{1118}; $rh3^{2}$	This manuscript	N/A
$rh4^{1}$ outcrossed to w^{1118}	w^{1118}; $rh4^{1}$	This manuscript	N/A
$rh6^{G}$ outcrossed to w^{1118}	w^{1118}; $rh6^{G}$	This manuscript	N/A
$Gq\alpha^{1}$ outcrossed to w^{1118}	*w1118*; $Gq\alpha^{1}$	This manuscript	N/A
CantonS	*CantonS* (Janelia Farm)	This manuscript	N/A
CantonS outcrossed to w^{1118}	*CantonS*	This manuscript	N/A
OregonR	*OregonR*	Bloomington *Drosophila* Stock Center	BL25125
Trp^{P365}	*w[*]; trp[P365]*	Bloomington *Drosophila* Stock Center	BL9044
GMR-GAL4	*w[*]; P{w[+mC]=GAL4-ninaE.GMR}12*	Bloomington *Drosophila* Stock Center	BL1104
Elav-GS-GAL4	*y[1] w[*]; P{w[+mC]=elav-Switch.O}GSG301*	Bloomington *Drosophila* Stock Center	BL43642
Trpl-GAL4	*w;trpl-GAL4/Tm6B,Tb*	Bloomington *Drosophila* Stock Center	BL52274
Trpl-GAL4; $GAL80^{ts}$	*w;trpl-GAL4/CyO;tub-*$GAL80^{ts}$	This manuscript	N/A
CLK^{out}	*w*;[ClkOUT]*	Bloomington *Drosophila* Stock Center	BL56754
cry^{01}	*cry[01]*	Our lab	N/A
cry^{02}	*cry[02]*	Our lab	N/A
cry^{B}	*crybaby*	Our lab	N/A
tim^{01}	*tim[01]*	Our lab	N/A
UAS-*Clk*	UAS-*Clk*	Gift from the lab of Paul E. Hardin	N/A
UAS-csChrimson (optogenetic)	*w[1118] P{y[+t7.7] w[+mC]=20XUAS-IVS-CsChrimson.mVenus}attP18*	Bloomington *Drosophila* Stock Center	BL55134
UAS-CLKΔ1	*w[*]; P{w[+mC]=UAS-Clk.Delta}1*	Bloomington *Drosophila* Stock Center	BL36318
UAS-CLKΔ1 outcrossed to w^{1118}	*w[*]; P{w[+mC]=UAS-Clk.Delta}1*	This manuscript	N/A
UAS-CLKΔ2	*w[*]; P{w[+mC]=UAS-Clk.Delta}865*	Bloomington *Drosophila* Stock Center	BL36319
Gβ76c-RNAi	*y[1] v[1]; P{y[+t7.7] v[+t1.8]=TRiP.JF03127}attP2*	Bloomington *Drosophila* Stock Center	BL28507
tsp42Ej-RNAi (*sunglasses*)	*y[1] v[1]; P{y[+t7.7] v[+t1.8]=TRiP.JF03325}attP2/TM3, Sb[1]*	Bloomington *Drosophila* Stock Center	BL29392
retinin-RNAi	*y[1] sc[*] v[1] sev[21]; P{y[+t7.7] v[+t1.8]=TRiP.HMC04693}attP40*	Bloomington *Drosophila* Stock Center	BL57389
ATPα-RNAi	*y[1] sc[*] v[1] sev[21]; P{y[+t7.7] v[+t1.8]=TRiP.HMS00703}attP2*	Bloomington *Drosophila* Stock Center	BL28073
nrv2-RNAi	*y[1] v[1]; P{y[+t7.7] v[+t1.8]=TRiP.JF03081}attP2*	Bloomington *Drosophila* Stock Center	BL28666
nrv3-RNAi	*y[1] v[1]; P{y[+t7.7] v[+t1.8]=TRiP.HMJ22547}attP40*	Bloomington *Drosophila* Stock Center	BL60367
RNAi-cnt (BDSC)	*y[1] sc[*] v[1] sev[21]; P{y[+t7.7] v[+t1.8]=VALIUM20-mCherry}attP2*	Bloomington *Drosophila* Stock Center	BL35785
arr1-RNAi	*w1118; P{GD11744}v22196/TM3*	Vienna *Drosophila* Resource Center	v22196
RNAi-cnt (VDRC)	*y,w[1118];P{attP,y[+],w[3`]*	Vienna *Drosophila* Resource Center	v60100

Supplementary Table 2. *Drosophila* strains used in this study.

Supplementary References

1 Chaudhari, A., Gupta, R., Makwana, K. & Kondratov, R. Circadian clocks, diets and aging. *Nutr Healthy Aging* **4**, 101-112, doi:10.3233/NHA-160006 (2017).

2 Sato, S. *et al.* Circadian Reprogramming in the Liver Identifies Metabolic Pathways of Aging. *Cell* **170**, 664-677 e611, doi:10.1016/j.cell.2017.07.042 (2017).

3 Eckel-Mahan, K. L. *et al.* Reprogramming of the circadian clock by nutritional challenge. *Cell* **155**, 1464-1478, doi:10.1016/j.cell.2013.11.034 (2013).

4 Cho, E. *et al.* AMP-Activated Protein Kinase Regulates Circadian Rhythm by Affecting CLOCK in Drosophila. *J Neurosci* **39**, 3537-3550, doi:10.1523/JNEUROSCI.2344-18.2019 (2019).

5 Ramanathan, C. *et al.* mTOR signaling regulates central and peripheral circadian clock function. *PLoS Genet* **14**, e1007369, doi:10.1371/journal.pgen.1007369 (2018).

6 Bae, S. A., Fang, M. Z., Rustgi, V., Zarbl, H. & Androulakis, I. P. At the Interface of Lifestyle, Behavior, and Circadian Rhythms: Metabolic Implications. *Front Nutr* **6**, 132, doi:10.3389/fnut.2019.00132 (2019).

7 Kuintzle, R. C. *et al.* Circadian deep sequencing reveals stress-response genes that adopt robust rhythmic expression during aging. *Nat Commun* **8**, 14529, doi:10.1038/ncomms14529 (2017).

8 Zhang, M. *et al.* Dysregulated metabolic pathways in age-related macular degeneration. *Sci Rep* **10**, 2464, doi:10.1038/s41598-020-59244-4 (2020).

9 Vallee, A., Lecarpentier, Y., Vallee, R., Guillevin, R. & Vallee, J. N. Circadian Rhythms in Exudative Age-Related Macular Degeneration: The Key Role of the Canonical WNT/beta-Catenin Pathway. *Int J Mol Sci* **21**, doi:10.3390/ijms21030820 (2020).

10 Baba, K. & Tosini, G. Aging Alters Circadian Rhythms in the Mouse Eye. *J Biol Rhythms* **33**, 441-445, doi:10.1177/0748730418783648 (2018).

11 Felder-Schmittbuhl, M. P. *et al.* Ocular Clocks: Adapting Mechanisms for Eye Functions and Health. *Invest Ophthalmol Vis Sci* **59**, 4856-4870, doi:10.1167/iovs.18-24957 (2018).

12 Kawashima, M. *et al.* Calorie restriction (CR) and CR mimetics for the prevention and treatment of age-related eye disorders. *Exp Gerontol* **48**, 1096-1100, doi:10.1016/j.exger.2013.04.002 (2013).

13 Baba, K., Ribelayga, C. P., Michael Iuvone, P. & Tosini, G. The Retinal Circadian Clock and Photoreceptor Viability. *Adv Exp Med Biol* **1074**, 345-350, doi:10.1007/978-3-319-75402-4_42 (2018).

14 Partch, C. L., Green, C. B. & Takahashi, J. S. Molecular architecture of the mammalian circadian clock. *Trends Cell Biol* **24**, 90-99, doi:10.1016/j.tcb.2013.07.002 (2014).

15 Baba, K. *et al.* Removal of clock gene Bmal1 from the retina affects retinal development and accelerates cone photoreceptor degeneration during aging. *Proc Natl Acad Sci U S A* **115**, 13099-13104, doi:10.1073/pnas.1808137115 (2018).

16 Sawant, O. B. *et al.* The Circadian Clock Gene Bmal1 Controls Thyroid Hormone-Mediated Spectral Identity and Cone Photoreceptor Function. *Cell Rep* **21**, 692-706, doi:10.1016/j.celrep.2017.09.069 (2017).

17 Fu, Y. & Yau, K. W. Phototransduction in mouse rods and cones. *Pflugers Arch* **454**, 805-819, doi:10.1007/s00424-006-0194-y (2007).

18 Montell, C. Drosophila visual transduction. *Trends Neurosci* **35**, 356-363, doi:10.1016/j.tins.2012.03.004 (2012).

19 Do, M. T. & Yau, K. W. Intrinsically photosensitive retinal ganglion cells. *Physiol Rev* **90**, 1547-1581, doi:10.1152/physrev.00013.2010 (2010).

20 Owens, L. *et al.* Effect of circadian clock gene mutations on nonvisual photoreception in the mouse. *Invest Ophthalmol Vis Sci* **53**, 454-460, doi:10.1167/iovs.11-8717 (2012).

21 Shieh, B. H. Molecular genetics of retinal degeneration: A Drosophila perspective. *Fly (Austin)* **5**, 356-368, doi:10.4161/fly.5.4.17809 (2011).

22 Nash, T. R. *et al.* Daily blue-light exposure shortens lifespan and causes brain neurodegeneration in Drosophila. *NPJ Aging Mech Dis* **5**, 8, doi:10.1038/s41514-019-0038-6 (2019).

23 Baik, L. S., Recinos, Y., Chevez, J. A. & Holmes, T. C. Circadian modulation of light-evoked avoidance/attraction behavior in Drosophila. *PLoS One* **13**, e0201927, doi:10.1371/journal.pone.0201927 (2018).

24 Pittendrigh, C. S. Temporal organization: reflections of a Darwinian clock-watcher. *Annu Rev Physiol* **55**, 16-54, doi:10.1146/annurev.ph.55.030193.000313 (1993).

25 Nippe, O. M., Wade, A. R., Elliott, C. J. H. & Chawla, S. Circadian Rhythms in Visual Responsiveness in the Behaviorally Arrhythmic Drosophila Clock Mutant Clk(Jrk). *J Biol Rhythms* **32**, 583-592, doi:10.1177/0748730417735397 (2017).

26 Storch, K. F. *et al.* Intrinsic circadian clock of the mammalian retina: importance for retinal processing of visual information. *Cell* **130**, 730-741, doi:10.1016/j.cell.2007.06.045 (2007).

27 Organisciak, D. T., Darrow, R. M., Barsalou, L., Kutty, R. K. & Wiggert, B. Circadian-dependent retinal light damage in rats. *Invest Ophthalmol Vis Sci* **41**, 3694-3701 (2000).

28 Ferrucci, L. & Fabbri, E. Inflammageing: chronic inflammation in ageing, cardiovascular disease, and frailty. *Nat Rev Cardiol* **15**, 505-522, doi:10.1038/s41569-018-0064-2 (2018).

29 Fougere, B., Boulanger, E., Nourhashemi, F., Guyonnet, S. & Cesari, M. Chronic Inflammation: Accelerator of Biological Aging. *J Gerontol A Biol Sci Med Sci* **72**, 1218-1225, doi:10.1093/gerona/glw240 (2017).

30 Kounatidis, I. *et al.* NF-kappaB Immunity in the Brain Determines Fly Lifespan in Healthy Aging and Age-Related Neurodegeneration. *Cell Rep* **19**, 836-848, doi:10.1016/j.celrep.2017.04.007 (2017).

31 Du, Y., Veenstra, A., Palczewski, K. & Kern, T. S. Photoreceptor cells are major contributors to diabetes-induced oxidative stress and local inflammation in the retina. *Proc Natl Acad Sci U S A* **110**, 16586-16591, doi:10.1073/pnas.1314575110 (2013).

32 Yang, Y., Hou, L., Li, Y., Ni, J. & Liu, L. Neuronal necrosis and spreading death in a Drosophila genetic model. *Cell Death Dis* **4**, e723, doi:10.1038/cddis.2013.232 (2013).

33 Srinivasan, N. *et al.* Actin is an evolutionarily-conserved damage-associated molecular pattern that signals tissue injury in Drosophila melanogaster. *Elife* **5**, doi:10.7554/eLife.19662 (2016).

34 Kondratov, R. V., Kondratova, A. A., Gorbacheva, V. Y., Vykhovanets, O. V. & Antoch, M. P. Early aging and age-related pathologies in mice deficient in BMAL1, the core componentof the circadian clock. *Genes Dev* **20**, 1868-1873, doi:10.1101/gad.1432206 (2006).

35 Boomgarden, A. C. *et al.* Chronic circadian misalignment results in reduced longevity and large-scale changes in gene expression in Drosophila. *BMC Genomics* **20**, 14, doi:10.1186/s12864-018-5401-7 (2019).

36 Mazzotti, D. R. *et al.* Human longevity is associated with regular sleep patterns, maintenance of slow wave sleep, and favorable lipid profile. *Front Aging Neurosci* **6**, 134, doi:10.3389/fnagi.2014.00134 (2014).
37 Kumar, S., Mohan, A. & Sharma, V. K. Circadian dysfunction reduces lifespan in Drosophila melanogaster. *Chronobiol Int* **22**, 641-653, doi:10.1080/07420520500179423 (2005).
38 Libert, S., Bonkowski, M. S., Pointer, K., Pletcher, S. D. & Guarente, L. Deviation of innate circadian period from 24 h reduces longevity in mice. *Aging Cell* **11**, 794-800, doi:10.1111/j.1474-9726.2012.00846.x (2012).
39 Inokawa, H. *et al.* Chronic circadian misalignment accelerates immune senescence and abbreviates lifespan in mice. *Sci Rep* **10**, 2569, doi:10.1038/s41598-020-59541-y (2020).
40 Patel, S. A., Chaudhari, A., Gupta, R., Velingkaar, N. & Kondratov, R. V. Circadian clocks govern calorie restriction-mediated life span extension through BMAL1- and IGF-1-dependent mechanisms. *FASEB J* **30**, 1634-1642, doi:10.1096/fj.15-282475 (2016).
41 Shen, J. & Tower, J. Effects of light on aging and longevity. *Ageing Res Rev* **53**, 100913, doi:10.1016/j.arr.2019.100913 (2019).
42 Hori, M., Shibuya, K., Sato, M. & Saito, Y. Lethal effects of short-wavelength visible light on insects. *Sci Rep* **4**, 7383, doi:10.1038/srep07383 (2014).
43 Katewa, S. D. *et al.* Peripheral Circadian Clocks Mediate Dietary Restriction-Dependent Changes in Lifespan and Fat Metabolism in Drosophila. *Cell Metab* **23**, 143-154, doi:10.1016/j.cmet.2015.10.014 (2016).
44 McLay, L. K., Green, M. P. & Jones, T. M. Chronic exposure to dim artificial light at night decreases fecundity and adult survival in Drosophila melanogaster. *J Insect Physiol* **100**, 15-20, doi:10.1016/j.jinsphys.2017.04.009 (2017).
45 Hughes, M. E., Hogenesch, J. B. & Kornacker, K. JTK_CYCLE: an efficient nonparametric algorithm for detecting rhythmic components in genome-scale data sets. *J Biol Rhythms* **25**, 372-380, doi:10.1177/0748730410379711 (2010).
46 Love, M. I., Huber, W. & Anders, S. Moderated estimation of fold change and dispersion for RNA-seq data with DESeq2. *Genome Biol* **15**, 550, doi:10.1186/s13059-014-0550-8 (2014).
47 Wijnen, H., Naef, F., Boothroyd, C., Claridge-Chang, A. & Young, M. W. Control of daily transcript oscillations in Drosophila by light and the circadian clock. *PLoS Genet* **2**, e39, doi:10.1371/journal.pgen.0020039 (2006).
48 Hall, H. *et al.* Transcriptome profiling of aging Drosophila photoreceptors reveals gene expression trends that correlate with visual senescence. *BMC Genomics* **18**, 894, doi:10.1186/s12864-017-4304-3 (2017).
49 Abruzzi, K. C. *et al.* Drosophila CLOCK target gene characterization: implications for circadian tissue-specific gene expression. *Genes Dev* **25**, 2374-2386, doi:10.1101/gad.174110.111
10.1101/gad.178079.111 (2011).
