# Supplementary material for: Dietary restriction and the transcription factor clock delay eye aging to extend lifespan in Drosophila Melanogaster
Source: Nat Commun. 2022 Jun 7;13:3156. doi: 10.1038/s41467-022-30975-4 (PMC9174495; doi:10.1038/s41467-022-30975-4)
Supplement: Supplementary file 3 — Description of Additional Supplementary Files [file 41467_2022_30975_MOESM3_ESM.pdf]

Description of Additional Supplementary files

File name: Supplementary Data 1

Description: AL and DR circadian transcriptome analyses in wild-type and circadian mutant flies. These files contain JTK_CYCLE P and Q value statistics, accompanied gene-ontology enrichment terms/scores for AL and DR circadian transcriptomes, circadian acrophase analyses, and differential gene-expression analyses for Canton-S and tim01 mutants. P values were calculated with hypergeometric distribution (findGO.pl, HOMER) with no adjustment for multiple-hypothesis testing.

File name: Supplementary Data 2.

Description: Description of fly lines utilized in this study. A detailed description of the fly strains, their genotypes, the figures in which they appear (a, main figures and b, supplementary figures), their outcrossed status, our experimental rational for using each strain, and their perceived strengths and weaknesses.

File name: Supplementary Data 3.

Description: Additional nCLK-Δ1 RNA-Seq analyses. Included in these files are normalized count reads generated by DEseq2 across all experimental groups and replicates from the nCLK-Δ1 RNA-Seq. The normalized expression counts across all samples for the gene-ontology terms "Deactivation of rhodopsin mediated signaling" and "Antimicrobial humoral response" (corresponding to Fig. 2b and 4b) are also reported.

File name: Supplementary Data 4.

Description: Cross-comparison of wild-type circadian transcriptome and nCLKΔ1 RNA-Seq analyses. These files include gene- ontology enrichment terms/scores for genes that are circadian in wild-type heads (from Kuintzle et al., 2017, GSE81100) and downregulated in nCLK-Δ1 heads. Geneontology enrichment scores and accompanied P values were calculated with the findGO.pl within the HOMER software package. P values for circadian rhythmicity were calculated with ARSER.

File name: Supplementary Data 5.

Description: Positive phototaxis responses and statistics. These files report detailed statistics (t test and 2way ANOVA) for the positive phototaxis experiments performed. Supplementary Data 6. Electroretinogram analyses and statistics. These files include detailed t test statistics for the ERG assays performed at day 14 and 21 in nCLK-Δ1 flies.

File name: Supplementary Data 7.

Description: Experimental materials. A detailed description of the materials, primer sequences, software packages, and commercial assays used in this study.

File name: Supplementary Data 8.

Description: nCLK-Δ1 hemolymph mass-spec analysis. These files contain the proteins identified and quantification of differential expression comparing proteomic profiles between nCLK-Δ1 and control hemolymph. Differential protein expression analysis was performed using a paired t test and P values were

adjusted for multiple testing. Peptides were identified at Q ≤0.01%, significantly changed proteins were accepted at a 5% FDR (Q ≤0.01). Enriched bioprocesses are also included for significantly up- or downregulated proteins. P values were calculated with hypergeometric distribution (findGO.pl, HOMER) with no adjustment for multiple-hypothesis testing.

File name: Supplementary Data 9.

Description: Analyses of transcriptional responses to light. These files report gene-ontology enrichment terms/scores for genes that are differentially expressed in wild-type fly heads in response to being housed in 12:12h LD vs constant dark (from Wijnen et al., 2006, GSE3842) and correspond to Supplementary Fig. 5e-f. Differential expression changes in response to light were calculated by averaging the differences in expression at each timepoint from the available circadian time-course microarray and comparing expression between flies housed in LD vs. DD. Individual genes were scored as significantly differentially expressed by performing a Student's t test (paired, twotailed) with P≤0.05. P values were calculated with hypergeometric distribution (findGO.pl, HOMER) with no adjustment for multiplehypothesis testing.

File name: Supplementary Data 10.

Description: Survival analyses. These files report lifespan statistics (Log-Rank and Hazard Ratios) and group sizes (n) for the survival analyses performed. The Log-rank (Mantel-Cox) test was used to determine statistical significance comparing average lifespan curves from a minimum of two independent lifespan replicates. Hazard Ratios (log-rank) were also utilized to determine the probability of death across genotypes, lighting conditions, and diet.

File name: Supplementary Data 11.

Description: Bioinformatic pipeline for identification of eye-specific and DRsensitive CLK-output genes Gbeta76c, retinin, and sun. These files provide the filtered genelists that correspond to the bioinformatic filtering steps performed in Fig. 6a-d and Supplementary Fig. 9a. Age P values (nonadjusted) were originally reported in Kuintzle et al., 2017 [16] and were calculated with Cuffdiff comparing transcript expression at day 5 to day 55 in wildtype Canton-S heads. P values for circadian rhythmicity were calculated with ARSER. Differential expression P values (non-adjusted) comparing genes from control and nCLK-Δ1 flies and between different diets were calculated with DESeq2.
