# Supplementary material for: Dietary restriction and the transcription factor clock delay eye aging to extend lifespan in Drosophila Melanogaster
Source: Nat Commun. 2022 Jun 7;13:3156. doi: 10.1038/s41467-022-30975-4 (PMC9174495; doi:10.1038/s41467-022-30975-4)
Supplement: Supplementary file 15 — Reporting Summary [file 41467_2022_30975_MOESM15_ESM.pdf]

nature research

Corresponding author(s): Pankaj Kapahi

Last updated by author(s): April 18, 2022

Reporting Summary

Nature Research wishes to improve the reproducibility of the work that we publish. This form provides structure for consistency and transparency in reporting. For further information on Nature Research policies, see our Editorial Policies and the Editorial Policy Checklist.

Statistics

For all statistical analyses, confirm that the following items are present in the figure legend, table legend, main text, or Methods section.

n/a	Confirmed	
☐	☒	The exact sample size (*n*) for each experimental group/condition, given as a discrete number and unit of measurement
☐	☒	A statement on whether measurements were taken from distinct samples or whether the same sample was measured repeatedly
☐	☒	The statistical test(s) used AND whether they are one- or two-sided<br>*Only common tests should be described solely by name; describe more complex techniques in the Methods section.*
☐	☒	A description of all covariates tested
☐	☒	A description of any assumptions or corrections, such as tests of normality and adjustment for multiple comparisons
☐	☒	A full description of the statistical parameters including central tendency (e.g. means) or other basic estimates (e.g. regression coefficient) AND variation (e.g. standard deviation) or associated estimates of uncertainty (e.g. confidence intervals)
☐	☒	For null hypothesis testing, the test statistic (e.g. *F*, *t*, *r*) with confidence intervals, effect sizes, degrees of freedom and *P* value noted<br>*Give P values as exact values whenever suitable.*
☒	☐	For Bayesian analysis, information on the choice of priors and Markov chain Monte Carlo settings
☒	☐	For hierarchical and complex designs, identification of the appropriate level for tests and full reporting of outcomes
☒	☐	Estimates of effect sizes (e.g. Cohen's *d*, Pearson's *r*), indicating how they were calculated

Our web collection on statistics for biologists contains articles on many of the points above.

Software and code

Policy information about availability of computer code

Data collection	1.) Data collection was performed using a combination of Microsoft Excel (version 16.58) and GraphPad Prism software (version 9).
Data analysis	2.) Data analysis was performed with either GraphPad Prism (version 9) or R software (4.0.2). Circadian statistics were generated with JTK_CYCLE algorithm (v3.0). Differential gene expression analysis for microarray were performed using the LIMMA software package (version 3.34.5) for R (4.0.2). Fastq sequences were trimmed using Trimmomatic (version 0.36). Differential gene expression analysis for RNA seq data was performed using the DEseq2 R package (galaxy version 2.11.40.7+galaxy1), HISAT2 (Galaxy Version 2.2.1+galaxy1), featureCounts (Galaxy Version 2.0.1+galaxy2). Heatmaps were generated with Heatmap2 (Galaxy Version 3.0.1). Gene ontology analyses were performed with the findGO.pl package of HOMER (v4.11). UCSC genome browser visualizations were created with makeUCSCfile from HOMER (v4.11).

Data

Policy information about availability of data

All manuscripts must include a data availability statement. This statement should provide the following information, where applicable:

- Accession codes, unique identifiers, or web links for publicly available datasets
- A list of figures that have associated raw data
- A description of any restrictions on data availability

The time-course microarray data and accompanied JTK_CYCLE statistics that support the findings in this study have been deposited in the Gene Expression Omnibus [77] with the GSE158286 accession code https://www.ncbi.nlm.nih.gov/geo/query/acc.cgi?acc=GSE158286. The RNAseq data and accompanied differential expression analyses that support the findings in this study have been deposited to GEO with the GSE158905 accession code https://www.ncbi.nlm.nih.gov/geo/query/acc.cgi?acc=GSE158905. The mass spectrometric raw data generated in this study have been deposited at https://massive.ucsd.edu/ProteoSAFe/dataset.jsp?accession=MSV000086781 with the MassIVE ID MSV000086781; it is also available at ProteomeXchange with the ID PXD023896. Additional mass spectrometric details from DIA and DDA acquisitions, such as protein identification and quantification details are available at the repositories (including all generated Spectronaut and Protein Pilot search engine files). The wildtype circadian gene expression data analyzed in this study are available in the GEO database under accession code GSE81100 https://www.ncbi.nlm.nih.gov/geo/query/acc.cgi?acc=GSE81100. The LD vs DD gene expression data analyzed in this study are available in the GEO database under accession code GSE3842 https://www.ncbi.nlm.nih.gov/geo/query/acc.cgi?acc=GSE3842. Data on photoreceptor enriched genes analyzed in this study are available in the GEO database under accession code GSE93782 https://www.ncbi.nlm.nih.gov/geo/query/acc.cgi?acc=GSE93782. Source data are provided with this paper. All other data supporting the findings of this study are included within the manuscript and supplemental information/data files.

Field-specific reporting

Please select the one below that is the best fit for your research. If you are not sure, read the appropriate sections before making your selection.

☒ Life sciences ☐ Behavioural & social sciences ☐ Ecological, evolutionary & environmental sciences

For a reference copy of the document with all sections, see nature.com/documents/nr-reporting-summary-flat.pdf

Life sciences study design

All studies must disclose on these points even when the disclosure is negative.

Sample size	Sample size was chosen using our lab's experience and the standards in the field of circadian biology, and Drosophila aging and behavioral science. For circadian time-course microarray experiments we sampled 3 biologically independent pools of RNA from flies at a 4 hour sampling density for a full circadian time-course which is standard for the field (https://www.ncbi.nlm.nih.gov/pmc/articles/PMC5692188/). The majority of lifespan experiments were performed with approximately 200 flies per experimental replicate which is approximately double the standard of 100 flies in the field (https://www.ncbi.nlm.nih.gov/pmc/articles/PMC3582515/). The phototaxis experiments were performed with large cohorts of flies (20 per biological replicate) (https://www.ncbi.nlm.nih.gov/pmc/articles/PMC3359294/). ERG experiments were performed with greater than 3 biological replicates which is standard for this procedure. Power calculations were not performed prior to experimentation.
Data exclusions	No data were excluded from our analyses.
Replication	Each Drosophila behavioral experiment consisted of 8 biological replicates per condition (n=160), and was repeated three times (i.e. three independent cohorts), totaling ~480 animals per condition. Each lifespan consisted of 200 animals per condition and was independently repeated 2-3 times as indicated. RT-qPCR data represent the average of three biological replicates (i.e. three independent RNA extractions from 20-60 animals as indicated). All replicate experiments were successful and all data were included in our analyses.
Randomization	Animals of the proper genotype were determined, and then randomly sorted into relevant conditions (i.e., AL or DR food, with or without drug).<br>The data collected in this study were purely quantitative and did not require judgment or scoring from the investigators analyzing the data.
Blinding	

Reporting for specific materials, systems and methods

We require information from authors about some types of materials, experimental systems and methods used in many studies. Here, indicate whether each material, system or method listed is relevant to your study. If you are not sure if a list item applies to your research, read the appropriate section before selecting a response.

Materials & experimental systems

n/a	Involved in the study	
☒	☐	Antibodies
☒	☐	Eukaryotic cell lines
☒	☐	Palaeontology and archaeology
☐	☒	Animals and other organisms
☒	☐	Human research participants
☒	☐	Clinical data
☒	☐	Dual use research of concern

Methods

n/a	Involved in the study	
☒	☐	ChIP-seq
☒	☐	Flow cytometry
☒	☐	MRI-based neuroimaging

Animals and other organisms

Policy information about studies involving animals; ARRIVE guidelines recommended for reporting animal research

Wild animals	We did not use wild animals in this study.
Field-collected samples	We did not use field-collected samples for this study.
Ethics oversight	This work did not require ethics oversight since our experiments were performed exclusively with fruit flies.

Note that full information on the approval of the study protocol must also be provided in the manuscript.

Laboratory animals Laboratory animals: *Drosophila melanogaster* mated females were used in this study. *Canton-S* and *Tim*^{01} mutant flies (11 days old) were used for the time-course microarray experiment. nCLK-Δ1 flies (11 days old) were used for the RNA-Seq. experiment. The following strains (6, 10, 14, 18, and 22 days old) were used in phototaxis experiments: nCLK-Δ1, prCLK-cnt, prCLK-Δ1, prCLK-OE, GMR-GAL4>RNAi, GMR-GAL4>*Gbeta76c*-RNAi, GMR-GAL4>*Retinin*-RNAi, GMR-GAL4>*Sun* -RNAi, *Canton-S* , *Oregon-R* , CLK^{OUT}, nCLK-Δ2, *Cry01* , *Cry02* , *CryB* , GMR-GAL4>*ATPalpha* -RNAi, GMR-GAL4>*arr1* -RNAi, GMR-GAL4>*nrv2* -RNAi, GMR>GAL4>*nrv3* -RNAi, Spa-GAL4>RNAi-cnt, Spa-GAL4>*ATPalpha* -RNAi. The follow strains (6, 10, 14, 18, and 25 days old) were used in ERG experiments: nCLK-Δ1, prCLK-cnt, prCLK-Δ1, prCLK-OE. The follow strains (6 and 14 days old) were used in tangential eye sections experiments: prCLK-cnt, prCLK-Δ1, prCLK-OE. The following strains (11 days old) were used in RT-PCR experiments: nCLK-Δ1, nCLK-Δ2, *w*^{1118}, *ninaE*^{17}, *rh3*^{2}, *rh4*^{1}, *rh6*^{G}. The following strains (6-days old to death) were used for lifespan analyses: nCLK-Δ1, nCLK-Δ2, *w1118*, *ninaE*^{17}, *rh3*^{2}, *rh4*^{1}, *rh6*^{G}, *Gq*^{1}, prCLK-cnt, prCLK-Δ1, prCLK-OE, GMR-GAL4>RNAi, GMR-GAL4>*Gbeta76c*-RNAi, GMR-GAL4>*Retinin*-RNAi, GMR-GAL4>*Sun*-RNAi, *Canton-S*, csChrimson, TRP^{365}, GMR-GAL4>*ATPalpha*-RNAi, GMR-GAL4>*arr1*-RNAi, GMR-GAL4>*nrv2*-RNAi, GMR>GAL4>*nrv3*-RNAi, Spa-GAL4>RNAi-cnt, Spa-GAL4>*ATPalpha*-RNAi. nCLK-Δ1 flies at 18 days of age were used in the hemolymph mass-spectrometry experiment.
