# Supplementary material for: Dietary restriction and the transcription factor clock delay eye aging to extend lifespan in Drosophila Melanogaster
Source: Nat Commun. 2022 Jun 7;13:3156. doi: 10.1038/s41467-022-30975-4 (PMC9174495; doi:10.1038/s41467-022-30975-4)
Supplement: Supplementary file 16 — Source Data [file 41467_2022_30975_MOESM16_ESM.zip › Source_Data/PR_Sections.pdf]

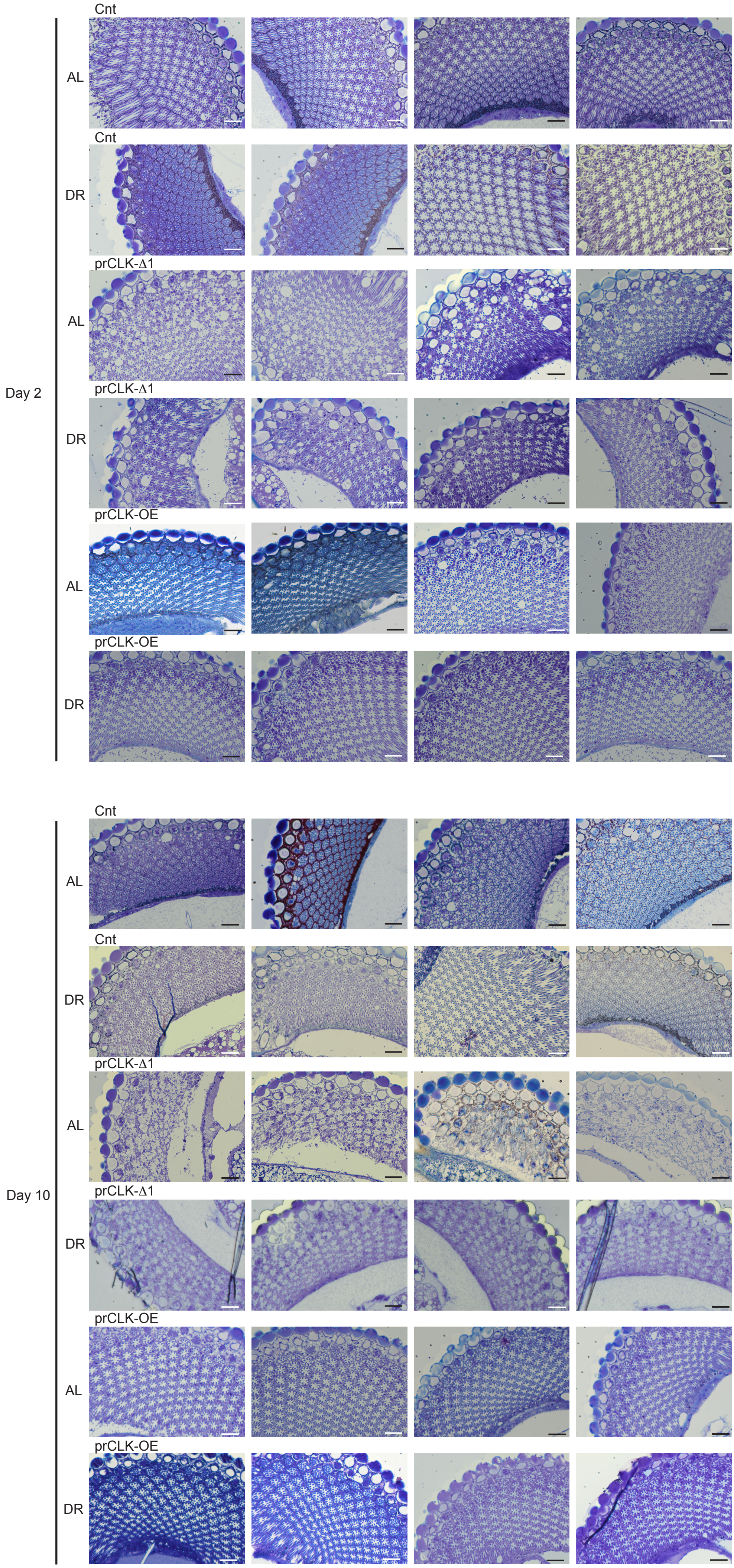
Day 2
Cnt
AL
Cnt
DR
prCLK-Δ1
AL
prCLK-Δ1
DR
prCLK-OE
AL
prCLK-OE
DR
Day 10
Cnt
AL
Cnt
DR
prCLK-Δ1
AL
prCLK-Δ1
DR
prCLK-OE
AL
prCLK-OE
DR
